# Can you repeat the question? Paradata as a lens to understand respondent experience answering cognitively demanding, sensitive questions

**DOI:** 10.1371/journal.pone.0252512

**Published:** 2021-06-07

**Authors:** Suzanne O. Bell, David Bishai

**Affiliations:** Department of Population, Family and Reproductive Health, Johns Hopkins Bloomberg School of Public Health, Baltimore, MD, United States of America; University of Dhaka: Dhaka University, BANGLADESH

## Abstract

Survey researchers hope that respondents will provide high-quality data, but evidence suggests that social desirability bias may be commonplace. Social desirability can lead to significant underreporting or overreporting of sensitive behaviors. With better understanding of the cognitive processes that respondents use to prepare and deliver their responses, survey designers could hope to minimize social desirability bias or at least detect settings that lessen its impact. The primary objective of this study was to use survey paradata to understand the psychology of responding to certain types of survey questions. More specifically, we sought to determine how emotional triggering can alter response latencies to cognitively demanding and sensitive survey questions on induced abortion, which is underreported. We hypothesize that having had a prior abortion might lengthen response times to an indirect question about abortion among respondents who have experienced this sensitive reproductive outcome as they hesitate in deciding whether and how to respond to the question. Data come from a representative survey of 6,035 reproductive age women in Rajasthan, India. We used list experiment question active screen time paradata in conjunction with responses from direct questions on abortion to assess our hypothesis. Our final model was a multivariate linear regression with random effects at the level of the interviewer, including adjustments for respondent, community, and interviewer characteristics to estimate within-respondent effects. Results suggest that women who reported an abortion on the direct abortion questions took 5.11 (95% CI 0.21, 10.00) seconds longer to respond to the list experiment treatment list compared to the control list in comparison to women who did not report an abortion on the direct abortion questions. This study demonstrates the additional insights gained when focusing on response latencies to cognitively demanding questions involved in the measurement of sensitive behaviors.

## Background

Relatively little is known regarding how respondents manage the cognitive and emotional demands of answering sensitive survey questions. Survey researchers hope that respondents will provide high-quality data, but evidence suggests that social desirability bias may be commonplace. Social desirability can lead to significant underreporting or overreporting of sensitive behaviors [1]. With better understanding of the cognitive processes that respondents use to prepare and deliver their responses, survey designers could hope to minimize social desirability bias or at least detect settings that lessen its impact. Survey paradata–which capture screen time, keystrokes, and survey navigation [2]–may provide insights into better understanding this phenomenon.

Exactly *how* social desirability influences survey response processes and response latencies is a source of ongoing debate. One perspective views social desirability in surveys as a deliberate, utility-maximizing behavior whereby respondents take more time to respond to questions that elicit this emotional response [3,4]. In one study of this phenomenon, an investigator assessed the effect of social desirability pressure by conducting a series of experiments that randomized respondents to receive standard survey instructions or instructions that increased the level of social desirability pressure [5,6]. Examining the resulting survey response latencies paradata, findings from multiple experiments indicated that those who received the heightened social desirability condition responded significantly more slowly than the standard condition. The author interprets these results as evidence of an internal response editing mechanism whereby participants mentally determine their response but ultimately provide a different, less stigmatizing answer when faced with social desirability pressure [5].

One the other hand, some research indicates that respondents under heightened social pressure may instead fail to engage in certain stages of the response process and thus provide a *quicker* response, an example of strong satisficing whereby respondents seek answers that are simply satisfactory or acceptable, instead of accurate, in order to reduce psychological expenditure [7–9]. Under this scenario, social desirability triggers an automatic behavior whereby respondent’s answer automatically in a manner that conforms with social norms [4,10]. In one study, investigators randomized participants to complete a personality test and found that those randomly instructed to provide answers that were indicative of a certain type of person (e.g. good, bad) took significantly less time to respond to questions than those who received standard instructions and answered on behalf of themselves [9]. The interpretation is that those who received the faking instructions did not engage in the retrieval process and thus could respond more quickly. Additional more recent research has observed similar findings for very desirable traits specifically [10]. However, much of the response latency research was conducted in the context of small studies with narrow respondent populations and examined attitude or beliefs. Further research is needed to understand how question difficulty and sensitivity with regard to behaviors impact respondents on population-based surveys as these aspects of survey design affect the quality of the resulting data. The current study will address this gap in the literature.

The primary objective of this study was to use survey paradata to understand the psychology of responding to certain types of survey questions. More specifically, we sought to determine how emotional triggering can alter response latencies, or the total time taken to respond to a survey question, to cognitively demanding and sensitive survey questions on induced abortion. The emotional triggering stems from the stigma surrounding abortion and the incentive to not disclose one’s experience with the behavior in order to avoid potential psychological discomfort and judgment from the interviewer. Survey active screen time paradata present an additional tool for researchers assessing evidence regarding the performance of more cognitively challenging indirect questions on sensitive topics and the quality of the resulting data. There can be multiple reasons for response delays. However, we focused on the respondents’ need to calculate emotional consequences from revealing a stigmatized behavior. Below we first describe the list experiment, an indirect method for asking about sensitive topics that we used in our study. We then review existing literature on social desirability pressure in surveys in order to explain the specific motivation for our hypothesis about how emotional consequences would affect response latencies to the list experiment questions on abortion.

### The list experiment

To address underreporting regarding a sensitive behavior of interest, we used an indirect method known as a list experiment which reduces the social desirability pressures of direct questioning [11]. Further details on the method and our experience implementing it are published elsewhere [12]. The standard list experiment randomizes individuals to either the treatment or control group. The control group is read a list of non-sensitive items, while the treatment group is read the same list, plus the sensitive item. Interviewers then ask respondents to report *how many* of the items they have ever experienced, not *which* ones, without directly mentioning each item (See Table 1 for an example.) The double list experiment, which we utilized, is a modification that allows for every respondent to receive a treatment version of one list and a control version of another list, thus each respondent serves as control and treatment within the sample [13]. The “how many” question adds the enumerative burden of counting events as well as scanning memory to assess whether they occurred. In theory, because the respondent reports “X events on that list have happened to me” they will feel more open in offering an accurate response as compared to having to disclose “Yes, event Y happened to me”. However, existing evidence on whether the list experiment increases data validity by reducing the incentive to underreport has been mixed [14–19]. The hypothesis we describe below explores one of the mechanisms that could explain the list experiment’s failure to produce a more valid estimate than direct report of a sensitive behavior [12].

**Table 1:** Double list experiment directions, questions, and items.

**Directions**	Now I would like to ask you a set of questions that are in a different format. I will read you a list of items and I just want you to tell me how many apply to you, not which ones. Wait until I have read the entire list to you, then tell me how many you have experienced. Your answer might be "none", "one", "two", "three", and so on, not telling me specifically which ones you have experienced. It may help to count the number of items you have experienced on your fingers. I can turn to the side to give you privacy so I don’t know which specific items you have experienced. Again, your answer for these questions should be a number, not “yes” or “no” for individual items or a list of the specific items. Do you understand?		
**Example question**	First let’s do an example. I am going to read you a list of foods and then I will tell you how many I have eaten in the last week. • Goat • Rice • Chicken • Potatoes My answer would be (your numeric answer). Now please tell me how many of the foods you have eaten in the previous week, not which ones. Remember, your answer should only be a number. Again, the list is: •Goat •Rice •Chicken •Potatoes		
**Actual question**	Now please tell me how many of the following health experiences you have ever had, not which ones:		
**Items**	**List A**	**List B**	**Population prevalence**^1^
Item 1	Had a menstrual period	Used a sanitary pad during a menstrual period	High
Item 2	Used contraceptive injections	Used a female condom	Low
Item 3^2^	Had an abortion	Had an abortion	Unknown
Item 4	Visited a health facility or camp	Been visited by an anganwadi, ASHA, or other community health worker	High
Item 5	Had a C-section	Taken an ambulance to a hospital	Low

^1^Lifetime prevalence estimated directly or inferred based on estimates of item prevalence in recent period (e.g. last 12 months) from prior data collected by DHS and PMA2020.

^2^Sensitive item; only added in treatment version of the list.

### Emotional effects on respondent strategizing and mental editing

Respondents may decide to edit responses, factoring in the response options and social desirability pressure. Mentioning a sensitive topic in a survey question can trigger a respondent’s awareness to emotional facets of the topic and the need to be strategic about the ways a subsequent response may or may not be socially desirable. It is challenging to distinguish between respondents who have a sincere but exaggerated or incorrect perception of themselves and respondents who are deliberately seeking to maintain a favorable impression by inaccurately responding to items [1]. Paulhus (1984) calls these social desirability biases self-deception and impression management, respectively [20]. One way to empirically examine the presence of social desirability, and potentially the type of social desirability bias, is through examination of response latencies.

Response latencies, or the time a respondent takes to answer a question, have been used widely in survey research as a way to understand cognitive processes that underlie survey responses and the strengths of attitudes [21,22]. Researchers interpret response latencies as a measure of item accessibility, or the relative ease with which an attitude (or behavior) comes to a respondent’s mind when asked about it in a survey question [21]. But they may also signal the influence of social desirability. While longer response latencies are generally interpreted as an indication of cognitive effort, editing answers in a socially desirable manner for impression management purposes is a specific theoretical assumption that could explain respondents taking longer to respond to sensitive questions than similar non-sensitive items [20].

Empirical research supports this idea of a deliberate mental editing process, where greater social desirability concerns are associated with greater response latencies [5,6,10]. For potentially affecting topics, this difference in response latency may be a result of a personal emotional response that the question content triggers. It may also be the case that sensitive topics raise the need for mental calculations and strategizing that has to take place in this last stage of responding as the participant calculates the social consequences of reporting a given answer. While anybody responding to a question that includes the word “abortion” might take longer than responding to questions without sensitive words, response latencies may be even longer for women who actually had an abortion because of a need to edit their answers based on social calculations; this is the group for whom a question on abortion is sensitive. For those who have not had an abortion, their answer would be quick and not elicit the same emotional response.

Other theoretical and empirical work suggests that this assumption of deliberate response editing may be more nuanced and dependent on the circumstances of the survey and the respondent characteristics. Researchers have demonstrated that whether a person engages in a deliberate, slow response mode or an automatic, quick response mode in the context of social desirability pressure may depend on the item desirability and its degree of salience [4,10]. Specifically, investigators observed an interaction between these features, with respondent’s who have a strong need for social approval in the context of a very desirable item responding *quickly* while the more deliberate response mode emerged in the context of highly undesirable items [4,10].

Since our work examines a highly salient sensitive behavior, instead of a sensitive attitude that a respondent may not have previously considered, and because the item desirability is uniformly and strongly in the negative direction, we believe this scenario will engender a deliberate response mode. We thus hypothesize that having had a prior abortion might lengthen response latencies to the treatment list among respondents who have experienced this sensitive reproductive outcome. We believe the net effect of editing and lengthening of response latencies will overwhelm any shortening of response latencies caused by satisficing at earlier stages of the response process. In conjunction with separately reported information on whether the woman has previously had an abortion, the structure of the list experiment offers an opportunity to assess the impact on response latency of asking about a sensitive item for women who we know have experienced the outcome. For women who have had an abortion, there might be hesitation when deciding whether and how to give a numerical response to this question.

In order to achieve the study objectives, we used the response latencies paradata associated with implementation of a double list experiment using smart phones. Specifically, we used the active screen time associated with the list experiment treatment and control questions to assess response patterns that may provide information regarding the psychological experience of answering cognitively demanding and potentially affecting survey questions. We describe the specific questions, variables, and analyses below.

## Methodology

### Data

Data come from Performance Monitoring and Accountability 2020 (PMA2020) data collection activities in Rajasthan, India. The Indian Institute of Health Management and Research (IIHMR) conducted the data collection, with technical assistance provided by researchers from the Bill & Melinda Gates Institute for Reproductive Health at the Johns Hopkins Bloomberg School of Public Health (JHSPH). The sampling strategy was based on a probabilistic multi-stage cluster sampling design with probability proportional to size used to select enumeration areas (EA) with urban/rural strata and regions as the sampling domains. PMA2020 conducts repeated cross-sectional surveys every 6 to 12 months in participating countries using female interviewers, most of whom resided in or near survey EAs. In Round 1 of data collection in Rajasthan, resident interviewers mapped and listed the 147 selected EAs; the same 147 EA sampling frames were subsequently used in Round 2, which was when we included the list experiment questions. In Round 2, 35 households were randomly sampled from each EA. Interviewers invited sampled households to participate in a brief household survey. Interviewers then requested all eligible women, i.e. those age 15 to 49, to participate in an interview related to reproductive health. Prior to administering the survey, interviewers asked for consent from all participants. Interviewers conducted all surveys face-to-face in either English or a Hindi translation of the questionnaire. Interviewers also used oral translations of local dialects when necessary to aide comprehension. The response rate (calculated using the Demographic and Health Survey approach) for the initial household questionnaire and the subsequent female surveys were both 98.3% [23]. The Institutional Review Boards (IRBs) at the JHSPH and IIHMR provided ethical approval of the study protocol.

We included the list experiment and direct abortion questions in the female questionnaire. We randomized half of the respondents to receive control list A (i.e. not including the sensitive item) followed by treatment list B (i.e. including the sensitive item). The other half of the respondents received control list B followed by treatment list A. Thus, every respondent received the control version of one list first and the treatment version of the other list second. In order to limit women’s ability to determine the intent behind the list experiment questions, we placed them in the first section of the survey before any other reproductive health questions had been asked, including the direct abortion questions. Prior to answering the list experiment questions, interviewers read list experiment instructions to the respondents, asked an example list experiment question with food items to familiarize the respondent with the question format, and then asked the randomly assigned control and treatment lists. The specific language and items for these questions are in Table 1. We describe the selection and piloting of the control items elsewhere [12]. We embedded the direct abortion questions in the reproductive history section later in the survey. These questions first asked if the woman had ever had a pregnancy that did not end in a live birth, and if she replied “yes”, inquired as to how the pregnancy ended–miscarriage, stillbirth, or abortion.

In addition to the household and female survey data, paradata collected via log files on the smart phones interviewers used to conduct the surveys recorded the active screen time–measured in milliseconds–for each question. As such, the paradata provided the approximate response latencies for each survey question, enabling investigation of potential response biases. The response latency data for a given question include both the time for the interviewer to read the question and the time for the woman to respond. To examine whether interviewer characteristics impacted question response latency, we also utilized the interviewer characteristics data, which came from a survey administered to interviewers following Round 2 data collection.

### Analysis

The key hypothesis relies on comparing response time differences within women on different parts of the survey. We hypothesize that ΔT(T,C) = (T_Ti_-T_Ci_) will depend on women’s prior history of abortion where T_Ti_ is time (in seconds) spent on question list experiment treatment list T by respondent “*i*”, T_Ci_ is time spent on list experiment control list C by the same respondent “*i*”, and history of abortion is the exposure of interest, which comes from woman’s direct report of this experience on a later survey question. The use of within person differences in list experiment response times rather than between respondent differences avoids introducing unobservable person specific confounders of response time that could be correlated with abortion. This particular list experiment we are studying has an attractive feature of having all respondents answer a control list experiment question that does *not* include the “had an abortion” item as well as a treatment list experiment question of comparable design that *does* include the “had an abortion” item (i.e. a double list experiment). For this reason comparing ΔT(treatment versus control) between women who disclosed having had an abortion in a direct question and women who did not could help to detect the potential effects of editing.

We first conducted univariate analyses (weighted to account for the complex survey design) to examine the distribution of respondent and interviewer characteristics, as well as the distribution of response times for the list experiment questions. We confirmed that the difference in response times were normally distributed, thus we did not need to create a logarithmic version of the variable in order to meet the linear regression assumption of normality. We recoded outliers greater than three standard deviations above the mean response latencies for the raw control and treatment list experiment variables, which impacted 1.8% of responses for each. For the main analyses we did not drop “speeders” from the raw response latency variables given we wanted to capture those potentially engaging in the more rapid automatic response mode. We then conducted bivariate analyses using adjusted Wald statistics to test for response time differences on abortion related questions by socioeconomic characteristics.

To assess the editing effects hypothesis, the exposure of interest was whether the respondent reported an abortion via the direct abortion questions and the outcome of interest was the difference between treatment versus control list response time, ΔT(T,C). Due to limitations in the interpretation of passive response latency, we leveraged the double list experiment design to examine within-person differences in list experiment response time. We modeled the multivariate analysis using ordinary least squares (OLS), however we also tried models controlling for fixed effects and random effects at the level of the interviewer as we were concerned about interviewer effects on abortion reporting and list experiment question implementation. We used Hausman tests to determine which model was most appropriate given the observed data. Models included adjustment for respondents’ age, marital status, education, wealth quintile, caste, religion, residence, parity, and whether they were acquainted with the resident interviewer; these are level-1 variables. OLS and random effects models also included the resident interviewers’ age, education, whether ever married, whether the interviewer thought abortion was legal under any circumstances, and whether the interviewer thought the list questions were difficult to implement; these are level-2 variables. For the final model (Table 4), which was a random effects generalized least squares model assuming exchangeable correlation structure, we applied survey weights that account for the complex survey design and non-response, representing the inverse probability of selection for an individual respondent. We also used the Taylor linearization method to estimate robust standard errors that account for clustering among respondents within the same EA. Additionally, we used cluster mean centered respondent (level-1) variables, thus, coefficients for respondent characteristics represent the within interviewer difference in response time associated with each level-1 variable (Begg and Parides 2003). Lastly, we conducted sensitivity analyses dropping “speeders” who responded to list experiment questions very quickly (less than 3 seconds or less than 5 seconds) to determine whether our results changed. We conducted all analyses in Stata version 15 and assessed statistical significance using an alpha of 0.05 [24].

## Results

In total, 6,035 women age 15 to 49 from selected households completed the female survey. Although “no response” or “do not know” was a valid response option for interviewers to enter, the response rates for the initial direct abortion question (regarding past non-live birth), the question about how the non-live birth ended, and the list experiment treatment list (with the abortion item) were all 99.9% (results not shown).

We present the sample characteristics in Table 2. On average, women were 29 years old, and the majority (75.7%) were currently married or cohabiting. A high proportion of women had never attended school (36.8%), were of other backward castes (39.2%), were Hindu (85.3%), or resided in rural areas (64.2%). Nearly one-third (31.1%) of women were nulliparous, while 36.1% had 1 to 2 children and 24.7% had 3 to 4 children; only 8.2% had 5 or more children. Reported lifetime experience of abortion was 3.5% via the direct questions and 1.8% via the double list experiment (Table 2).

**Table 2:** Socioeconomic characteristics of Rajasthani women age 15 to 49^1^.

	Total	
	%	N^2^
Mean age (SE)	28.9 (0.16)	6,035
Marital status		
Currently married/cohabiting	75.7	4,557
Divorced or separated/widowed	2.7	162
Never married	21.6	1,302
School		
Never attended	36.8	2,221
Primary	24.3	1,469
Secondary	17.6	1,059
Higher or postgraduate	21.3	1,285
Wealth		
Poorest	16.5	997
Second poorest	17.5	1,056
Middle	19.7	1,186
Second wealthiest	21.5	1,295
Wealthiest	24.9	1,500
Caste of household head		
Scheduled caste	22.3	1,346
Scheduled tribe	17.3	1,042
Other backward caste	39.2	2,362
General	21.2	1,279
Religion of household head		
Hindu	85.3	5,148
Muslim	13.3	801
Other	1.4	86
Residence		
Rural	64.2	3,874
Urban	35.8	2,160
Parity		
0	31.1	1,873
1–2	36.1	2,177
3–4	24.7	1,487
5+	8.2	493
Abortion (direct questions)		
No	96.5	5,823
Yes	3.5	211
Abortion (list questions)	1.8	6,035
Total	100.0	6,035

^1^Estimates and Ns weighted.

^2^Sample sizes inside categories does not always add to 6,035 due to missing data.

Response time for the direct question regarding past experience with a non-live birth averaged 11.0 seconds (SE 0.4) (Table 3). The list experiment related questions required much longer response latencies; on average 27.0 (SE 1.4) and 23.7 (SE 1.3) seconds for the control list experiment question and the treatment list experiment question, respectively (Table 3). Adjusted Wald test results revealed significant variation in response latencies for several questions within a number of socioeconomic characteristics. Older women consistently took longer to respond to the direct abortion and list experiment questions, as did women with more past births and who reported an abortion on the direct questions. In contrast, women who had never married tended to respond more quickly than women with other marital statuses.

**Table 3:** Average response latencies in seconds to list experiment questions in seconds among Rajasthani women age 15 to 49, by socioeconomic characteristics and question (N = 6,017)^1^.

	Ever non-live birth	Control list	Treatment list
	Response latency in seconds (SE)		
Age			
15–19	8.6 (0.5)***	20.1 (1.3)***	16.9 (1.2)***
20–29	11.2 (0.5)***	27.4 (1.5)***	24.3 (1.5)***
30–39	11.8 (0.5)***	29.4 (1.8)***	26.7 (1.6)***
40–49	12.0 (0.6)***	29.9 (1.9)***	24.9 (1.6)***
Marital status			
Currently married/cohabiting	11.8 (0.4)***	29.1 (1.5)***	25.8 (1.4)***
Divorced or separated/widowed	13.8 (1.5)***	30.2 (3.7)***	27.3 (3.2)***
Never married	8.1 (0.5)***	19.3 (1.2)***	15.9 (1.1)***
School			
Never attended	11.4 (0.5)	28.2 (1.6)	25.1 (1.5)
Primary	11.5 (0.6)	28.3 (1.9)	24.5 (1.6)
Secondary	10.4 (0.6)	25.5 (1.8)	21.8 (1.6)
Higher or postgraduate	10.4 (0.5)	24.7 (1.7)	21.8 (1.5)
Wealth			
Poorest	10.5 (0.5)	23.8 (2.0)	20.8 (1.6)
Second poorest	11.6 (0.6)	27.6 (1.8)	24.5 (1.6)
Middle	11.2 (0.6)	29.5 (2.0)	24.9 (1.8)
Second wealthiest	11.2 (0.6)	27.9 (2.0)	24.4 (1.8)
Wealthiest	10.6 (0.6)	26.0 (1.9)	23.4 (1.9)
Caste of household head			
Scheduled caste	10.8 (0.6)	25.6 (2.1)	23.4 (2.0)
Scheduled tribe	10.6 (0.5)	24.4 (2.2)	20.4 (2.2)
Other backward caste	11.2 (0.6)	28.6 (1.8)	24.8 (1.7)
General	11.3 (0.6)	27.7 (2.0)	24.6 (1.8)
Religion of household head			
Hindu	11.1 (0.4)	26.7 (1.4)	23.5 (1.3)
Muslim	10.4 (0.7)	28.9 (3.7)	24.5 (2.8)
Other	10.3 (1.3)	28.8 (5.4)	27.4 (5.2)
Residence			
Rural	11.3 (0.5)	27.3 (1.5)	23.7 (1.4)
Urban	10.5 (0.7)	26.5 (2.8)	23.6 (2.5)
Parity			
0	9.0 (0.4)***	20.4 (1.2)***	17.1 (1.1)***
1–2	11.7 (0.5)***	28.2 (1.7)***	24.9 (1.6)***
3–4	11.8 (0.5)***	31.0 (1.7)***	27.8 (1.7)***
5+	13.4 (0.8)***	34.9 (2.3)***	31.0 (1.8)***
Abortion (direct question)			
No	10.9 (0.4)	26.5 (1.4)***	22.9 (1.2)***
Yes	13.2 (1.4)	40.8 (3.2)***	45.1 (4.4)***
Total	11.0 (0.4)	27.0 (1.4)	23.7 (1.3)

^1^All estimates include weights accounting for complex survey design and non-response.

* denotes p<0.10,

** denotes p<0.05, and

*** denotes p<0.01; based on adjusted Wald test of null hypothesis that all response latencies were equal within the category.

Women who reported a past abortion via the direct questions took on average 45.1 (SE 4.4) seconds to respond to the treatment list that included “had an abortion” and women who reported no abortion took 22.9 (SE 1.2) seconds to respond (p<0.001) (Table 3). The Hausman test indicated the random effects model including interviewer characteristics was the preferred model (p = 0.89 in comparison to the fixed effects model), however the exposure variable was similar in magnitude and statistically significant in every model. In the final random effects model we found that adjusting for interviewer effects, women who reported an abortion on the direct abortion questions took 5.11 (95% CI 0.21, 10.00) seconds longer to respond to the list experiment treatment list compared to the control list in comparison to women who did not report an abortion on the direct abortion questions (Table 4). Results were similar in sensitivity analyses excluding “speeders” (i.e., those who responded in less than 3 seconds or 5 seconds). Women age 40 to 49 took 3.89 fewer seconds to respond (95% CI -7.04,-0.75) compared to women age 15 to 19 and women whose interviewer was age 20 to 29 responded 2.99 seconds faster (95% CI -5.84,-0.15) than those with interviewers age 15 to 19. Additionally, women from a scheduled tribe took 2.46 fewer seconds to respond (95% 95% CI -4.70,-0.22) than scheduled caste women (Table 4).

**Table 4:** Final random effects generalized least squares linear regression model of direct abortion reporting on within respondent difference in list experiment treatment and control list response latencies within interviewers^1^.

	β	95% CI
Direct abortion question response (reference no)		
Yes	5.11**	0.21,10.00
Age (reference 15–19)		
20–29	-1.31	-3.32,0.71
30–39	-2.03	-4.72,0.67
40–49	-3.89**	-7.04,-0.75
Marital status (reference currently married/cohabiting)		
Divorced/widowed	0.87	-2.91,4.65
Never married	-0.61	-3.44,2.21
Schooling (reference never attended)		
Primary school	-0.93	-2.26,1.33
Secondary school	-0.54	-2.96,1.89
Higher education	0.33	-2.10,2.76
Wealth quintile (reference poorest)		
Middle poorest	0.69	-1.39,2.77
Middle	0.5	-1.68,2.67
Middle wealthiest	1.8	-0.70,4.30
Wealthiest	1.39	-1.58,4.35
Caste (reference scheduled caste)		
Scheduled tribe	-2.46**	-4.70,-0.22
Other backward caste	-2.03	-4.55,0.49
Generate caste	0.00	-2.90,2.91
Religion (reference Hindu)		
Muslim	-1.01	-3.67,1.65
Other religion	-1.53	-6.19,3.14
Parity (reference 0)		
1–2	0.22	-1.99,2.43
3–4	1.11	-1.47,3.70
5+	3.19*	-0.19,6.58
Interviewer acquainted (reference no)		
Yes	-0.25	-2.00,1.51
Interviewer age (reference 15–19)		
20–29	-2.99**	-5.84,-0.15
30–39	-1.75	-5.48,1.98
40–49	-1.94	-9.91,6.04
Interviewer education (reference secondary or technical)		
University	-1.67	-3.75,0.40
Masters or doctoral	1.23	-0.96,3.42
Interviewer ever married (reference no)		
Yes	0.86	-1.87,3.60
Interviewer parity (reference 0)		
1–2	0.71	-1.61,3.02
3–4	3.06	-1.08,7.20
Interviewer thinks abortion illegal (reference no)		
Yes	0.11	-1.64,1.86
Constant	-1.84	-4.59,0.92
Rho	0.03	
AIC	47582.22	
N	5,298	

^1^Model applies survey weights that account for the complex sampling design and non-response, representing the inverse probability of selection. Used Taylor linearization method to calculate robust standard errors that account for clustering among respondents in the same EA.

* denotes p<0.10,

** denotes p<0.05, and *** denotes p<0.01.

## Discussion

This analysis presents new methods of leveraging the paradata that is increasingly collected in the course of survey implementation [25]. The paradata findings provide evidence of response editing or strategizing delays on the list experiment treatment question for women who reported a prior abortion on the direct abortion questions later in the survey. The delay may have been triggered when women who had had an abortion confronted the word “abortion” in the treatment list experiment question. The slower response could have been an affective process of recalled emotion and/or a process of rationally deliberating whether to edit a response to achieve social desirability. Since underreporting on direct abortion questions is substantial [26], these results on delayed response latencies are subject to misclassification of women who actually had abortions that they did not report with direct questioning. Thus, women who had an abortion but did not report an abortion with direct questioning later in the survey are being grouped together with women who never had an abortion.

The finding that women who reported experiencing a prior abortion via the direct questions took significantly longer to respond to a list experiment question that included the abortion item adds support to the idea that respondents engage in a strategizing and potential mental editing process whereby they are deciding internally how to respond when asked about sensitive items on surveys. Prior research has similarly demonstrated longer response latencies on survey questions that involve reporting socially undesirable attitudes or ideas in a controlled setting [4–6]. Our findings thus extend this previous literature to a real-world survey context asking about prior behaviors.

Although these indirect means of evaluating sources of bias in reporting sensitive items can be informative, the data collection activities were not explicitly designed to assess these hypotheses and have several limitations. Our findings offer only initial evidence regarding this response phenomenon, but alternative explanations cannot be ignored. Ideally, we would have also captured respondents’ perspective on the sensitivity and desirability of abortion and their need for social approval to examine these predictors’ relationship with response latencies in the models. Prior research has demonstrated an interaction between these factors but further empirical investigation with a range of traits and behaviors is needed [4]. The reference group of women who replied “no” to the direct abortion question includes women who did in fact have an abortion, thus the findings from this analysis may be biased as the reference group likely includes substantial misclassification. Additionally, the passively collected paradata are imperfect. Response latency is at best a proxy for capturing the respondent cognitive processes. Our response latency data include both the time it takes the interviewer to read the question and the time for the respondent to provide an answer; we are unable to distinguish between these times in the data. However, results adjust for interviewer characteristics via random and fixed effects and we leveraged the double list experiment design to model within-respondent effects, reducing the likelihood of biased results. It is unlikely that the use of passively collected response latencies resulted in qualitatively different results as research comparing active and passive approaches has found similar results regardless of method of response time data collection [21]. Lastly, to the extent that delays in responding are significant, the phone goes into an energy saving mode and this time is not captured in the response latencies paradata; the data do not indicate when the phone goes into energy saving mode so we cannot adjust for it. If these longer delays occurred systematically for certain types of women, this may have introduced bias.

Additional design limitations constrain our interpretation of the list experiment and associated paradata results. All women received the list experiment questions prior to the direct questions. However, putting the list experiment question after the direct questions may have shortened response latencies and improved reporting of the sensitive behavior if respondents had already been primed to think about abortion by earlier direct questions. A minority of respondents may have also viewed as sensitive those items that we thought were not sensitive. As these individuals should have been distributed evenly across the two groups of respondents, this should not have affected our sensitive item estimates.

Beyond these limitations, this study has a number of strengths. Our exposure of interest–abortion–was a highly salient sensitive behavior with a uniformly negative desirability that a respondent is likely to know whether they have experienced or not. This is in contrast to behaviors with more ambiguous desirability or a sensitive attitude or belief that some respondents may have never considered and for which they may be inclined to respond automatically in a socially desirable manner. Thus, the nature of our exposure strengthened our hypothesis suggesting the approval-motivated impression management presentation of social desirability in our study, even in the absence of additional details on respondents’ need for social approval. We tested our hypothesis using a large, population-based sample. As such, respondents encompass a diverse set of characteristics. We utilized paradata automatically captured via smart phone data collection, which enabled examination of a novel research question using secondary data. Additionally, we were able to adjust for individual, community, and interviewer characteristics and employ a rigorous analytic approach using within-person model with interviewer random effects in our effort to isolate the independent effect of the exposure variable for our hypothesis, thus strengthening the credibility of the findings.

Further research leveraging paradata from smart phone data collection in population-based surveys could improve our understanding of respondent psychological processes when answering sensitive survey questions, particularly those employing indirect methodologies that may be more challenging. Conducting this research with other sensitive topics would provide evidence as to whether our findings apply to stigmatizing behaviors more broadly. Specific to abortion, more research involving qualitative cognitive interviews would inform our knowledge of respondent interpretation of abortion-related question wording and the recall and retrieval process. Qualitative cognitive interviews would enable assessment of respondents’ ability to retrieve and enumerate past events in the case of list experiment questions. More broadly, studies of respondent psychology would have broad benefit to the vast array of researchers who depend on survey data. This study demonstrates the additional insights gained with regard to data quality concerns when focusing on response latencies to cognitively demanding questions involved in the measurement of sensitive behaviors.

## Supporting information

S1 File PMA2017 INR2 Rajasthan FQ v1 9Feb2018. (PDF)

S2 File Stage5 models 2021. (XLSX)

S3 File RJR2-PLOSONE article response time variables. (DTA)
