# Supplementary material for: Can you repeat the question? Paradata as a lens to understand respondent experience answering cognitively demanding, sensitive questions
Source: PLoS One. 2021 Jun 7;16(6):e0252512. doi: 10.1371/journal.pone.0252512 (PMC8183984; doi:10.1371/journal.pone.0252512)
Supplement: S1 File — (PDF) [file pone.0252512.s001.pdf]

Female Questionnaire
महिला प्रश्नावली

NO	QUESTIONS AND FILTERS	CODING CATEGORIES	Relevant If:
**IDENTIFICATION**			
001a	क्या आप ठीक घर के सामने है?<br>**Are you in the correct household?**<br>यह तस्वीर घर - परिवार प्रश्नावली के दौरान घर के सम्मुख खींची गई है।<br>**This is the picture of the front of the home taken during the Household Questionnaire.**<br>[ODK will display the photo attached to the linked Household Questionnaire]	हाँ/ Yes..1<br>नहीं/ No..0	Always<br>हमेशा
001b	**यदि नहीं है, तो सही घर-पारिवार के साक्षात्कार के लिए वापस जाएं।**<br>**RETURN TO INTERVIEW THE CORRECT HOUSEHOLD.**		001a=0
002	आपकी आई.डी.:<br>**Your ID:**<br>[Interviewer ID from Household Questionnaire]<br>क्या यह आपकी आई.डी. है ?<br>**Is this your ID?**	हाँ/ Yes..1<br>नहीं/ No..0	Always<br>हमेशा
	नीचे अपनी आई.डी. दर्ज करे<br>**Enter your ID below.**<br>कृपया अपनी आई.डी. दर्ज करे<br>*Please record your ID*	Interviewer's ID<br>साक्षात्कारकर्ता की आई.डी	002=0
003	वर्तमान दिनांक और समय।<br>**Current date and time.**<br>[ODK will display on screen]<br>क्या यह दिनांक और समय सही है?<br>**Is this date and time correct?**	हाँ/ Yes..1<br>नहीं/ No..0	Always<br>हमेशा
	सही दिनांक और समय रिकॉर्ड करें।<br>**Record the correct date and time.**	Day दिन | Month महीना | Year साल<br>Hours घंटे | Min मिनट | AM/PM	003=0

004a	निम्न जानकारी घर-परिवार प्रश्नावली से है। यह सुनिश्चित करने के लिए कृपया पुनः देख लें कि आप सही उत्तरदाता से साक्षात्कार कर रहे हैं<br>**The following information is from the Household Questionnaire. Please review to make sure you are interviewing the correct respondent.**<br>[ODK will display the District, Tehsil/Taluk, City/Town/Village, Enumeration Area, Structure Number, and Household Number entered into the Household Questionnaire linked to this Female Questionnaire.]<br>क्या ऊपर दी गई सूचना सही है?<br>**Is the above information correct?**	हाँ/ Yes ....1<br>नहीं/ No 0	Always<br>हमेशा
004b	सही घर-परिवार पर जायें या फिर ज़रूरत पड़ने पर घर-परिवार रोस्टर को अपडेट करें.<br>**GO TO THE RIGHT HOUSEHOLD OR UPDATE THE HOUSEHOLD ROSTER IF NEEDED**		004a=1
005	सुनिश्चित करें: मैं [Respondent's Name] का साक्षात्कार करने का प्रयास कर रही हुं। क्या यह सही है?<br>**CHECK: You should be attempting to interview [Respondent's Name]. Is that correct?**<br>*यदि यहाँ नाम की गलत वर्तनी (स्पेलिंग) हो तो, हाँ का चुनाव कीजिये और प्रश्न "011" में नाम को सही करें।*<br>*यदि सम्बन्धित व्यक्ति नहीं है तो आपके पास दो विकल्प है:*<br>*(1) बाहर निकलें और इस फार्म में किए गए परिवर्तनों को नज़रंदाज़ करें। सही फार्म खोलें।*<br>*या*<br>*(2) जिस व्यक्ति का नाम ऊपर प्रदर्शित (डिस्प्ले) हुआ था उसे ही ढूँढें और उसका साक्षात्कार करें।*<br>*If misspelled, select "yes" here and update the name in question "011."*<br>*If this is the wrong person, you have two options:*<br>*(1) exit and ignore changes to this form. Open the correct form.*<br>*Or*<br>*(2) find and interview the person whose name appears above.*	हाँ/ Yes ....1<br>नहीं/ No 0	Always<br>हमेशा

006	क्या उत्तरदाता आज साक्षात्कार के लिए मौजूद और उपलब्ध है?<br>**Is the respondent present and available to be interviewed today?**	**हाँ/** Yes .... 1<br>**नहीं/** No 0	Always<br>हमेशा
007	आप उत्तरदाता से कितनी अच्छी तरह से परिचित हैं?<br>**How well acquainted are you with the respondent?**	**बहुत अच्छी तरह से परिचित/** Very well acquainted .... 1<br>**अच्छी तरह से परिचित/** Well acquainted2<br>**अच्छी तरह से परिचित नहीं/** Not well acquainted .... 3<br>**परिचित नहीं/** Not acquainted 4	006 = 1
008	**as this respondent previously participated in PMA2020 surveys?**<br>क्या यह उत्तरदाता PMA2020 सर्वेक्षण में पहले भी भाग ले चुकी हैं?	हाँ/Yes.. 1<br>नहीं/No .... 0<br>नहीं जानती/Don't know.................... -88<br>कोई जवाब नहीं/No response .... -99	006=1

INFORMED CONSENT
सूचित सहमति
Find the woman between the ages of 15-49 associated with this Female Questionnaire. The interview must have auditory privacy. Read the following greeting:
इस महिला प्रश्नावली से जुड़ी 15-49 वर्ष के बीच की महिला को ढूंढे। साक्षात्कार के समय श्रवण गोपनीयता सुनिश्चित करें। निम्न लेख को पढ़ें:

009a	नमस्कार! मेरा नाम _____________ है और मै भारतीय स्वास्थ्य प्रबंध शोध विश्वविद्यालय और उनके स्थानीय सहयोगी स्वयंसेवी संगठन के लिए कार्य कर रही हूँ |मैं इसी/पास के गाँव की हूँ |हम एक स्थानीय सर्वे कर रहे है जिसके अंतर्गत महिलाओ के प्रजनन स्वास्थ्य से सम्बन्धित विभिन्न मुद्दों पर पूछताछ करेंगे जो की परिवार नियोजन और स्वास्थ्य सेवाओं के उन अंतरालों को पहचानने में मदद करेगा जो की उत्पादों की निरंतर उपलब्धता, जानकारी, सेवाओं, और गुणवत्ता को प्रभावित करते है | यह सर्वे पूरे राजस्थान में किया जा रहा है| प्रत्येक गणना क्षेत्र से 35 घरों को लिया गया है और इसी कड़ी में आपका घर चुना गया है|आपकी सहभागिता हमारे लिए बहुत ज़रूरी है लेकिन यह आपकी इच्छा पर निर्भर है, अगर आप मना भी करती है तो इसका कोई दुष्प्रभाव आप पर नहीं पड़ेगा| आपके द्वारा दी जाने वाली जानकारी सरकार को बेहतर स्वास्थ्य योजना बनाने में मदद करेगी| इसमें यूँ तो कोई खतरा नहीं है लेकिन व्यक्तिगत व सवेदनशील विषयों पर जानकारी साझा करने पर प्रतिभागियों को कुछ तनाव हो सकता है| इसका कोई सीधा लाभ भी आपको नहीं मिलेगा परन्तु हम यह मानते है कि यह अध्ययन हमें व हमारे जैसे कई समूहों को ऐसे कार्यक्रम विकसित करने में मदद करेगा जो कि राजस्थान व भारत में रह रहे समुदायों के लोगों का जीवन उन्नत बना सकेगा। अतः हमें उम्मीद है कि आप इस सर्वेक्षण में भाग लेंगी| जो कुछ भी जानकारी आप प्रदान करती हैं वह पूरी तरह से गोपनीय रखी जाएगी और हमारी सर्वेक्षण टीम के सदस्यों के अलावा अन्य किसी को नहीं दिखायी जायेगी| आकड़ो के विश्लेषण में हम आपके नाम का उपयोग नहीं करेंगे| आपके जवाबों से बने आकड़ो के डाटा बेस को पासवर्ड से सुरक्षित किये कम्प्यूटर और सर्वर पर ही रखा जायेगा| इस सर्वेक्षण में भाग लेना स्वैच्छिक है, और आप किसी भी सवाल का जवाब नहीं देना चाहती हैं तो बस मुझे बता दीजिये और मैं अगले प्रश्न पर चली जाउंगी या आप किसी भी समय साक्षात्कार रोक सकती है| सामान्यतः इस सर्वेक्षण को पूरा करने में 20 से 30 मिनिट लगते है। एक अनुसन्धान में भागीदार होने के नाते यदि इससे सम्बंधित कुछ प्रश्न है तो आप इसी समय मुझसे पूछ सकते हैं या भारतीय स्वास्थ्य प्रबंध शोध विश्वविद्यालय जयपुर राजस्थान के डॉ. अनूप खन्ना से 91.141.3924738 पर संपर्क कर सकते है।<br><br>Namaskar! My name is _____________ and I am working for the IIHMR University Jaipur in collaboration with local Partners. I belong to this/nearby village. We are conducting a local survey that asks women about various reproductive health issues, the result of which would help to identify gaps in family planning and health services that affect regular availability and quality of information, services, and products. Some of these questions have been added expressly for research purposes. This survey is conducted across Rajasthan. From every enumeration area 35 households have been randomly selected your house was one of those households selected. Though we would very much appreciate your participation in the survey, your refusal to take part in this survey will have no repercussions on you. Information provided by you would help us inform the government to better plan health services. There is no physical risk associated with participating in this survey; however, some participants may experience distress in sharing information on personal and sometimes sensitive topics. If you agree to take part in this study, it will not benefit you directly. However, we believe that information gathered in this study will help us and other groups like us to develop programs that will improve the lives of men and women in communities in Rajasthan and around India, thus we hope that you would take part in this survey. Whatever information you provide will be kept confidential and only our research team will have access to it. We will never use your name while analyzing the data. The tracking sheet and the database with your responses will be kept on password-protected computers and on a secure server. Participation in this study is entirely voluntary, if you do not wish to answer any question feel free to inform me and I would skip to the next question. You can choose not to participate at all or to end the interview at any point. The survey usually takes between 20 to 30 minutes to complete. If you have any questions about the study and your right as a research participant, you may ask me now or you may also contact Dr. Anoop Khanna at IIHMR University, in Jaipur, Rajasthan at +91-141-3924738.		
	सहमति प्रपत्र की एक प्रति दीजिए और उसे समझाइए - फिर पूछिए - क्या मैं अब साक्षात्कार शुरू कर सकती हूँ?<br><br>Provide a paper copy of the Consent Form to the respondent and explain it. Then, ask:<br>**May I begin the interview now?**	**हाँ**/ Yes ....1<br>**नहीं**/ No 0	006 =1

009b	उत्तरदाता के हस्ताक्षर<br>**Respondent's signature**<br>*कृपया उत्तरदाता से कहे की वह अपनी सहभागिता को सुनिश्चित करने के लिए सहमती पत्र की प्रति पर हस्ताक्षर करे या अपने अंगूठे की छाप दे तथा ओडीके फॉर्म पर भी सहमती देने के लिए इसी प्रक्रिया को दोहराएँ।*<br>*Please ask the respondent to sign the paper copy of the Consent Form or give a thumbprint on the paper copy of the Consent Form and do likewise in the ODK form in agreement of their participation.*	GATHER SIGNATURE:<br>बॉक्स चिन्हित करें<br>Check box: □	009a = 1
010	**Interviewer's ID:** [Interviewer ID from Household Questionnaire]<br>साक्षात्कारकर्ता की आई.डी.: [परिवार प्रश्नावली से लें]<br>*Mark your ID as a witness to the consent process.*<br>सहमति कि प्रक्रिया के गवाह के रूप में अपनी आई डी को दर्ज करें।		009a = 1
011	उत्तरदाता का पहला नाम।<br>**Respondent's first name**<br>[ODK will display the Respondent's name from linked Household Roster]<br>*अगर नाम सही नहीं है, तो आप यहाँ वर्तनी (स्पेलिंग) सही कर सकते हैं, लेकिन ठीक व्यक्ति का साक्षात्कार किया जाना चाहिए, जिसका नाम नीचे प्रदर्शित हुआ है।*<br>*You may correct the spelling here if it is not correct, but you must be interviewing the person whose name appears below.*		009a = 1
**Section 1 – Respondent's Background, Marital Status, and Household Characteristics**<br>भाग 1-उत्तरदाता की पृष्ठभूमि, वैवाहिक स्थिति, घर-परिवार की विशेषताएं<br>*अब मैं आपकी पृष्ठभूमि और सामाजिक आर्थिक स्थिति के बारे में पूछना चाहती हूं।*<br>*Now I would like to ask about your background and socioeconomic conditions.*			
101	आप किस महीने और साल में पैदा हुई थी?<br>घर-परिवार प्रश्नावली में उम्र [AGE] वर्ष है<br>**In what month and year were you born? The age in the household roster is [AGE].**	**महीना**/Month<br>**साल**/Year	009a = 1
102	अपने पिछले जन्मदिन पर आप कितने वर्ष की थीं?<br>**How old were you at your last birthday?**<br>14 वर्ष से अधिक की होनी चाहिए। 101 से अनुरूप होना चाहिए।<br>*Must be more than 14. Must agree with 101.*	**उम्र**/Age	009a = 1
103	आपने उच्चतम शिक्षा कहाँ तक प्राप्त की है?<br>**What is the highest level of school you attended?**	कोई शिक्षा प्राप्त नहीं की/Never Attended 0<br>प्राथमिक/Primary ....1<br>माध्यमिक/Secondary..........................2<br>उच्च माध्यमिक/Higher Secondary.........3<br>स्नातक या उससे ज्यादा/Graduate and Above...4<br>कोई जवाब नहीं/No response............-99	009a = 1

104	क्या आप वर्तमान में विवाहित है या शादी के रूप में एक पुरूष के साथ रह रही हैं?<br>**Are you currently married or living together with a man as if married?**<br>*गहराई से पूछें - यदि नहीं तो क्या उत्तरदाता तलाकशुदा, अलग या विधवा है।*<br>*Probe: If no, ask whether the respondent is divorced, separated, or widowed.*	हाँ, वर्तमान में विवाहित हूं/Yes, currently married..1<br>हाँ, एक आदमी के साथ रहती हूँ/Yes, living with a man ....2<br>विवाहित, परन्तु गौना नहीं हुआ/Yes, married, gauna not performed ....3<br>वर्तमान में तलाकशुदा / अलग/Not currently in union:<br>Divorced / separated........................4<br>विधवा/Not currently in union: Widow 5<br>नहीं, कभी साथ नहीं रही/No, never in union ....6<br>कोई जवाब नहीं/No response............-99	009a = 1
105	क्या एक या एक से अधिक बार आपकी शादी हुई है अथवा किसी पुरूष के साथ रही हैं?<br>**Have you been married or lived with a man only once or more than once?**	एक बार/Only once ....1<br>एक से अधिक बार/More than once........2<br>कोई जवाब नहीं/No response............-99	104 ≠ 6
106a	किस महीने और साल में आपने अपने पहले पति / साथी के साथ रहना शुरू किया?<br>**In what month and year did you start living with your FIRST husband / partner?**<br>*अगर कोई प्रतिक्रिया नहीं, Jan 2020 दर्ज करें*<br>*Enter Jan 2020 for no response.*	**महीना**/Month ____<br>**महीना**/Year ____	105=2
106b	जांचे:प्रश्न 106a में दिए गए उत्तर के अनुसार,उत्तरदाता अपने प्रथम विवाह के समय शायद 15 वर्ष या 15 वर्ष से कम आयु की थी क्या आपने 106a सही से दर्ज किया?<br>**CHECK: Based on the response you entered in 106a, the respondent was possibly 15 years old or younger at the time of her first marriage. Did you enter 106a correctly?**	**हाँ**/ Yes ....1<br>**नहीं**/ No 0	106a age at marriag e ≤15
106c	**RETURN TO 106A TO CORRECT BEFORE CONTINUING**<br>आगे बढ़ने से पहले, सुधार हेतु 106 a पर जाएँ।		106b = 0
107a	अब मैं आपसे पूछना चाहूंगी कि आपने अपने वर्तमान पति / साथी के साथ कब से रहना शुरू किया। कौन से महीने और साल से?<br>**Now I would like to ask about when you started living with your CURRENT or MOST RECENT husband / partner. In what month and year was that?**<br>*अगर कोई प्रतिक्रिया नहीं, Jan 2020 दर्ज करें*<br>*Enter Jan 2020 for no response.*	**महीना**/Month ____<br>**महीना**/Year ____	105 = 1, 2, or 3

107b	जांचें- 107a में आपके द्वारा दर्ज उत्तर के अनुसार उत्तरदाता अपनी वर्तमान/हाल ही में हुई शादी के समय 15 वर्ष या उससे कम की थी। क्या आपने 107a ठीक दर्ज किया है?<br>**CHECK: Based on the response you entered in 107a, the respondent was possibly 15 years old or younger at the time of her current or most recent marriage. Did you enter 107a correctly?**	**हाँ/** Yes ....1<br>**नहीं/** No 0	107a age at marriag e ≤15
107c	**RETURN TO 107A TO CORRECT BEFORE CONTINUING**<br>आगे बढ़ने से पहले, सुधार हेतु 107a पर जाएं।		107b = 0
108	क्या आपके पति / साथी की अन्य पत्नियाँ हैं या वे विवाहितों की तरह अन्य महिलाओं के साथ रहते हैं ?<br>**Does your husband / partner have other wives or does he live with other women as if married?**	हाँ/Yes...1<br>नहीं/No ....0<br>नहीं जानती/Don't know...................-88<br>कोई जवाब नहीं/No response ....-99	104 = 1 OR 2 OR 3
109	क्या आपके पति / साथी अभी आपके साथ रह रहे है या वह कहीं और रह रहे है?<br>**Is your husband / partner living with you now or is he staying elsewhere?**	उत्तरदाता के साथ रहते हैं/Living with respondent......................................1<br>कहीं और रह रहे हैं/Staying elsewhere ..2<br>कोई जवाब नहीं/No response ....-99	104 = 1 OR 2 OR 3

LST0	अब में आप से कुछ प्रश्न पुछुगी जिनका प्रारूप थोडा अलग है| अब मैं आपको एक सूची पढ कर सुनाउंगी आपको सिर्फ यह बताना है की उसमे से आप ने कितने अनुभव किये|यहाँ आप को उन वस्तूओ के नाम नही बताने| जितनी देर मैं सूचि पढ़ूं उतनी देर धैर्य रखे फिर मुझे उत्तर दे| आप का उत्तर "कोई नही","एक", "दो ", "तीन" इत्यादी हो सकते है, इसमें सिर्फ आप को नाम नही बताने हैं|आप यदि इन्हें अँगुलियों पर गिनना चाहे तो शायद आप आसानी से बता पाएंगे|आपकी सुविधा के लिए मैं आपकी ओर पीठ करके खड़ी हो सकती हूँ जिससे आपको थोड़ी निजता मिले|ध्यान रहे इन प्रश्नों का उत्तर आपको सिर्फ संख्या मे बताना है इनके नाम नहीं लेने है| <br>क्या आप समझ गयी ?<br><br>**Now I would like to ask you a set of questions that are in a different format. I will read you a list of items and I just want you to tell me how many apply to you, not which ones. Wait until I have read the entire list to you, then tell me how many you have experienced. Your answer might be "none", "one", "two", "three", and so on, not telling me specifically which ones you have experienced. It may help to count the number of items you have experienced on your fingers. I can turn to the side to give you privacy so I don't know which specific items you have experienced. Again, your answer for these questions should be a number, not "yes" or "no" for individual items or a list of the specific items.**<br><br>**Do you understand?**<br><br>*अगर उत्तरदाता ने खडी प्रश्नों का उत्तर सही सही(उत्तर सभी वस्तुओ या वस्तुओं की सूचि के लिए हाँ या ना के बजाये संख्या में होना चाहिए) दे दिया है तो अगले प्रश्न पर जाए| अगर उत्तरदाता कहती है की उसे समझ नही आया तो सूचि बद्ध प्रश्नों को पुन: समझाए और उसके बाद में उदाहरण के तौर पर किये गये खाद्य प्रश्नों को करें |*<br><br>*Proceed to the next question if the respondent says she understands. If the respondent says she does not understand, re-explain the list question instructions and then proceed to the example food question.*	**हाँ**/ Yes ....1<br>**नहीं**/ No 0	

LST1	यहाँ हम इसका एक उदाहरण देखेंगे । मैं आपके समक्ष वस्तुओं की एक सूची पढूँगी, उनमें से मैं बिना वस्तुओं के नाम लिए यह बताऊगी कि निम्न में से कितने पदार्थ पिछले हफ्ते मैंने खाए हैं:<br><br>-बकरा<br>-चावल<br>-चिकन<br>-आलू<br><br>यहाँ मेरा उत्तर (अंकों में आपका उत्तर है)अब मैं आपसे एक भिन्न प्रकार का प्रश्न पूछना चाहूँगी।अब कृपया मुझे बिना नाम लिए बतायें कि कितनी वस्तुएं आप पर लागु होती हैं। याद रखे उत्तर सिर्फ अंको में ही होना चाहिए।सूची इस प्रकार है:<br>-बकरा<br>-चावल<br>-चिकन<br>-आलू<br><br>**First let's do an example. I am going to read you a list of foods and then I will tell you how many I have eaten in the last week.**<br><br>**- Goat**<br>**- Rice**<br>**- Chicken**<br>**- Potatoes**<br><br>**My answer would be (your numeric answer). Now please tell me how many of the foods you have eaten in the previous week, not which ones. Remember, your answer should only be a number. Again, the list is:**<br><br>**- Goat**<br>**- Rice**<br>**- Chicken**<br>**- Potatoes**<br><br>*सूची को दोबारा पढ़ें।*<br><br>*यदि उत्तरदाता सूची में लिखी वस्तुओं का हाँ या ना में जवाब दे या उनका नाम लेकर बताने लगे तो उन्हें वहीं रोक दें ,निर्देश दुबारा समझाएं,व भोज्य सामग्री सूची का उदाहरण पुन:करें।*<br><br>*Read the list a second time.*<br><br>*If the participant answers with "yes" or "no" for individual items or a list of the specific items, STOP HER, re-explain the instructions, and do the food example again.*	कोई नहीं/None....................................0<br>कोई एक/One......................................1<br>कोई दो/Two ....2<br>कोई तीन/Three...................................3<br>कोई चार/Four.....................................4<br>नहीं जानती/Don't know....................-88<br>कोई जवाब नहीं/No response............-99	009a = 1

LST2	कृपया अब आप मुझे बताएं कि आपके अब तक निम्न में से कितने स्वास्थ्य संबंधित अनुभव रह चुकें हैंः<br>**Now please tell me how many of the following health experiences you have EVER had, not which ones:**<br>-मासिक धर्म रह चूका है/**Had your menstrual period**<br>-गर्भनिरोधक इंजेक्शन लिए हैं/**Used contraceptive injections**<br>***-अपनी इच्छा से कोई गर्भपात करवया/Had an induced abortion***<br>**-किसी स्वास्थ्य केंद्र या कैंप का दौरा किया है/Visited a health facility or camp**<br>**-बच्चा ऑपरेशन से हुआ है।/Had a c-section**<br>*सूची दोबारा पढ़कर सुनाएं।*<br>*यदि उत्तरदाता सूची में लिखी वस्तुओं का हाँ या ना में जवाब दे या उनका नाम लेकर बताने लगे तो उन्हें वहीं रोक दें, निर्देश दुबारा समझाएं।*<br>*Read the list a second time.*<br>*If the participant answers with "yes" or "no" for individual items or a list of the specific items, STOP HER and re-explain the instructions.*	कोई नहीं/None....................................0<br>कोई एक/One......................................1<br>कोई दो/Two.......................................2<br>कोई तीन/Three...................................3<br>कोई चार/Four.....................................4<br>सभी पाँच/*Five ....5*<br>नहीं जानती/Don't know....................-88<br>कोई जवाब नहीं/No response............-99	009a = 1

LST3	पुनः, कृपया आप मुझे बताएं कि आपके अब तक निम्न में से कितने स्वास्थ्य संबंधित अनुभव रह चुकें हैः<br><br>**Again, please tell me how many of the following health experiences you have EVER had, not which ones:**<br><br>**- माहवारी के दौरान सेनिटरी पैड/पैड का उपयोग किया है |/Used a sanitary pad during a menstrual period**<br>**- महिला कोंडोम का प्रयोग किया/Used a female condom**<br>***-अपनी इच्छा से कोई गर्भपात करवया/Had an induced abortion***<br>**- क्या आशा, आंगनवाडी कार्यकर्त्ता या अन्य सामुदायिक स्वास्थ्य कार्यकर्ता आपसे मिलने आई/Visited by an anganwadi worker, ASHA or other community health worker**<br>-अस्पताल जाने हेतु कोई एम्बुलेंस ली/**Took an ambulance to a hospital**<br><br>*सूची दोबारा पढ़कर सुनाएं।*<br><br>*यदि उत्तरदाता सूची में लिखी वस्तुओं का हाँ या ना में जवाब दे या उनका नाम लेकर बताने लगे तो उन्हें वहीं रोक दें, निर्देश दुबारा समझाएं|*<br><br>*Read the list a second time.*<br>*If the participant answers with “yes” or “no” for individual items or a list of the specific items, STOP HER and re-explain the instructions.*	कोई नहीं/None....................................0<br>कोई एक/One.....................................1<br>कोई दो/Two......................................2<br>कोई तीन/Three..................................3<br>कोई चार/Four....................................4<br>सभी पाँच/*Five*....................................*5*<br>नहीं जानती/Don’t know....................-88<br>कोई जवाब नहीं/No response............-99	009a = 1

LST4	अब मैं आपसे विशेषतः पिछले वर्ष के आपके अनुभव जानना चाहूँगी। कृपया बताएं कि आपके निम्न में से कितने स्वास्थ्य संबंधित अनुभव पिछले 12 महीनों के दौरान रह चुकें हैंः<br>**Now I would like to know about your experience with these items specifically in the previous year. Please tell me how many of the following health experiences you have had in the LAST 12 MONTHS, not which ones:**<br><br>-मासिक धर्म रह चूका है/**Had your menstrual period**<br>-गर्भनिरोधक इंजेक्शन लिए हैं/**Used contraceptive injections**<br>***-अपनी इच्छा से कोई गर्भपात करवया/Had an induced abortion***<br>**-किसी स्वास्थ्य केंद्र या कैंप का दौरा किया है/Visited a health facility or camp**<br>**-बच्चा ऑपरेशन से हुआ है|/Had a c-section**<br><br>*सूची दोबारा पढ़कर सुनाएं।*<br><br>*यदि उत्तरदाता सूची में लिखी वस्तुओं का हाँ या ना में जवाब दे या उनका नाम लेकर बताने लगे तो उन्हें वहीं रोक दें, निर्देश दुबारा समझाएं|*<br>*Read the list a second time.*<br>*If the participant answers with "yes" or "no" for individual items or a list of the specific items, STOP HER and re-explain the instructions.*	कोई नहीं/None....................................0<br>कोई एक/One......................................1<br>कोई दो/Two.......................................2<br>कोई तीन/Three...................................3<br>कोई चार/Four.....................................4<br>सभी पाँच/*Five* ....*5*<br>नहीं जानती/Don't know....................-88<br>कोई जवाब नहीं/No response............-99	009a = 1

LST5	पुनः, कृपया बताएं कि आपके निम्न में से कितने स्वास्थ्य संबंधित अनुभव पिछले 12 महीनों के दौरान रह चुकें हैं<br>**Again, please tell me how many of the following health experiences you have had in the LAST 12 MONTHS, not which ones:**<br><br>**- माहवारी के दौरान सेनिटरी पैड/पैड का उपयोग किया है |/Used a sanitary pad**<br>**- महिला कोंडोम का प्रयोग किया/Used a female condom**<br>***-अपनी इच्छा से कोई गर्भपात करवया/Had an induced abortion***<br>**- क्या आशा, आंगनवाडी कार्यकर्त्ता या अन्य सामुदायिक स्वास्थ्य कार्यकर्ता आपसे मिलने आई/Visited by an anganwadi worker, ASHA or other community health worker**<br>-अस्पताल जाने हेतु कोई एम्बुलेंस ली/**Took an ambulance to a hospital**<br><br>*सूची दोबारा पढ़कर सुनाएं।*<br><br>*यदि उत्तरदाता सूची में लिखी वस्तुओं का हाँ या ना में जवाब दे या उनका नाम लेकर बताने लगे तो उन्हें वहीं रोक दें, निर्देश दुबारा समझाएं|*<br>*Read the list a second time.*<br>*If the participant answers with "yes" or "no" for individual items or a list of the specific items, STOP HER and re-explain the instructions.*	कोई नहीं/None....................................0<br>कोई एक/One......................................1<br>कोई दो/Two.......................................2<br>कोई तीन/Three...................................3<br>कोई चार/Four.....................................4<br>सभी पाँच/*Five*....................................*5*<br>नहीं जानती/Don't know....................-88<br>कोई जवाब नहीं/No response............-99	009a = 1
**Section 2 – Reproduction, Pregnancy & Fertility Preferences**<br>भाग 2 - प्रजनन, गर्भावस्था और जननक्षमता प्राथमिकताएं			
200	अब मैं आपसे उन बच्चो के बारे में पूछना चाहूंगी जिन्हें आपने अपने जीवनकाल में जन्म दिया है। क्या आपने कभी किसी बच्चे को जन्म दिया है?<br>**Now I would like to ask about all the births you have had during your life. Have you ever given birth?**	हाँ/Yes..1<br>नहीं/No...0<br>कोई जवाब नहीं/No response............-99	009a=1
LCL_201	[illegible]?<br>**How many times have you given birth?**<br>[illegible], -99 [illegible] 0 [illegible]<br><br>*Enter -99 for no response. 0 is a possible answer.*	संख्या/Number ☐	200=1

201a	क्या आपके कोई ऐसे बेटे या बेटियाँ हैं जिन्हें आपने स्वयं जन्म दिया हो और वो वर्तमान में आपके साथ रह रहे हों?<br>**Do you have any sons or daughters to whom you have given birth who are now living with you?**	हाँ/Yes...1<br>नहीं/No ....0<br>कोई जवाब नहीं/No response ....-99	200=1
201b	कितने बेटे आपके साथ रहते हैं?<br>**How many sons live with you?**<br>उत्तर शून्य भी हो सकता है। कोई जवाब न देने पर -99 कोड डालें।<br>*Zero is a possible response. Enter -99 for No response.*	संख्या/Number ☐	201a = 1
201c	कितनी बेटियाँ आपके साथ रहती हैं?<br>**How many daughters live with you?**<br>उत्तर शून्य भी हो सकता है। कोई जवाब न देने पर -99 कोड डालें।<br>*Zero is a possible response. Enter -99 for No response.*	संख्या/Number ☐	201a = 1
202a	क्या आपके कोई ऐसे बेटे या बेटियाँ हैं जिन्हें आपने स्वयं जन्म दिया हो और वे जीवित हो लेकिन वे वर्तमान में आपके साथ नहीं रह रहे हों?<br>**Do you have any sons or daughters to whom you have given birth who are alive but do not live with you?**	हाँ/Yes...1<br>नहीं/No ....0<br>कोई जवाब नहीं/No response ....-99	200=1
202b	कितने बेटे ऐसे हैं जो जीवित हैं लेकिन आपके साथ नहीं रहते हैं?<br>**How many sons are alive but do not live with you?**<br>उत्तर शून्य भी हो सकता है। कोई जवाब न देने पर -99 कोड डालें।<br>*Zero is a possible response. Enter -99 for No response.*	संख्या/Number ☐	202a = 1
202c	कितनी बेटियाँ ऐसी हैं जो जीवित हैं लेकिन आपके साथ नहीं रहती हैं?<br>**How many daughters are alive but do not live with you?**<br>उत्तर शून्य भी हो सकता है। कोई जवाब न देने पर -99 कोड डालें।<br>*Zero is a possible response. Enter -99 for No response.*	संख्या/Number ☐	202a = 1

203a	क्या आपने कभी किसी ऐसे लड़के या लड़की को जन्म दिया है जो जीवित जन्मा/जन्मी हो लेकिन वह बाद में मृत हो गया/गयी हो?<br>**Have you ever given birth to a boy or girl who was born alive but later died?**<br><br>यदी जवाब नहीं है तो सुनिष्चित करने हेतु कुछ ऐसे पूछें◌ः ऐसा बच्चा जो जन्म के बाद (भले ही बहुत कम समय हेतु) रोया हो, हिला-डुला हो, या जिसने कोई आवाज़ की हो, या सांस लेने की कोशिश की हो, या जीवित होने का कोई भी संकेत दिया हो?<br>**IF NO, PROBE: Any baby who cried, who made any movement, sound, or effort to breathe, or who showed any other signs of life even if for a very short time?**	हाँ/Yes...1<br>नहीं/No...0<br>कोई जवाब नहीं/No response -99	009a=1
LCL_ 202	[illegible]?<br>**How many times have you given birth?**<br>[illegible], -99 [illegible] 0 [illegible]<br><br>*Enter -99 for no response. 0 is a possible answer.*	संख्या/Number	203a=1
203b	कुल कितने लड़कों की मृत्यू हुई हैं?<br>**How many boys have died?**<br><br>उत्तर शून्य भी हो सकता है। कोई जवाब न देने पर -99 कोड डालें।<br>*Zero is a possible response. Enter -99 for No response.*	संख्या/Number	203a = 1
203c	कुल कितनी लडकियों की मृत्यू हुई हैं?<br>**And how many girls have died?**<br>उत्तर शून्य भी हो सकता है। कोई जवाब न देने पर -99 कोड डालें।<br>*Zero is a possible response. Enter -99 for No response.*	संख्या/Number	203a = 1
CALC CEB	CALCULATE: NUMBER OF CHILDREN EVER BORN. ODK will automatically calculate the total number of children ever born from 201-203: (201b+201c+202b+202c+203b+203c)	संख्या/Number	200=1

204	**मइसे जोर से पढ़ें: मैं सुनिश्चित करना चाहती हूँ कि मैं सहीं हूँ, आपने अपने पूरे जीवन में कुल [LCL_201] बच्चों को जन्म दिया है उसमे से [CALC CEB] बेटे और बेटीयाँ है।**<br><br>**क्या यह सही है?**<br><br>**Just to make sure I have this right: you had a total of [LCL_201] birth(s) during your life, resulting in [CALC CEB] son(s) or daughter(s) born alive.**<br><br>**Is that correct?**	हाँ/Yes....................................1<br>नहीं/No....................................0	CALC CEB >0
205	आपने पहला जन्म कब दिया था?<br>**When was your FIRST birth?**<br><br>*सबसे पहले जीवित जन्म की तारीख रिकॉर्ड करें। तारीख जानने के लिए यदि आवश्यक हो तो यादगार घटनाओं से आगे या पीछे की गणना की जानी चाहिए। कोई जवाब नहीं देने की स्थिति में Jan 2020 दर्ज करें।*<br>*Please record the date of the FIRST birth. Date should be found by calculating forward or backward from memorable events if needed. Enter Jan 2020 for no response.*	**महीना**/Month [ ]<br>**महीना**/Year [ ]	LCL_201 >1 OR LCL_202 >1
206	अभी हाल ही में आपने कब जन्म दिया?<br>**When was your MOST RECENT birth?**<br><br>*सबसे हाल ही में हुए जीवित जन्म की तारीख रिकॉर्ड करें। तारीख जानने के लिए यदि आवश्यक हो तो यादगार घटनाओं से आगे या पीछे की गणना की जानी चाहिए। कोई जवाब नहीं देने की स्थिति में Jan 2020 दर्ज करें।*<br>*Please record the date of the MOST RECENT birth. The date should be found by calculating backwards from memorable events if needed.*<br>*Enter Jan 2020 for no response.*	**महीना**/Month [ ]<br>**महीना**/Year [ ]	LCL_201 >0 OR LCL_202 >0

207	आपने सबसे हाल ही में जन्मे शिशु से पहले कब जन्म दिया था ?<br>**When did you give birth before the most recent one?**<br><br>*हाल में दिए गए जन्म से पहले के जन्म की तारीख रिकॉर्ड करें। तारीख जानने के लिए यदि आवश्यक हो तो यादगार घटनाओं से आगे या पीछे की गणना की जानी चाहिए। कोई जवाब नहीं देने की स्थिति में Jan 2020 दर्ज करें।*<br>*Please record the date of the birth before the last. The date should be found by calculating backwards from memorable events if needed.*<br>*Enter Jan 2020 for no response.*	**महीना**/Month [ ]<br>**महीना**/Year [ ]	LCL_201 >2 OR LCL_202 >2
208a	क्या आपका सबसे छोटा शिशु / बच्चा जीवित है?<br>**Is your last baby / child still alive?**	हाँ/Yes....................1<br>नहीं/No....................0<br>नहीं जानती/Don't know..........-88<br>कोई जवाब नहीं/No response..........-99	LCL_201 > 0 OR LCL_202 >0
208b	आपके सबसे छोटे शिशु / बच्चे की मृत्यु कब हुई?<br>**When did your last baby / child die?**<br>*बच्चे की मृत्यु की तारीख रिकार्ड करें। तारीख जानने के लिए यदि आवश्यक हो तो यादगार घटनाओं से आगे या पीछे की गणना की जानी चाहिए। कोई जवाब नहीं देने की स्थिति में Jan 2020 दर्ज करें।*<br>*Please record the date of the child's death.*<br>*The date should be found by calculating backwards from memorable events if needed.*<br>*Enter Jan 2020 for no response.*	**महीना**/Month [ ]<br>**महीना**/Year [ ]	208a = 0
ABT 1	क्या कभी आपका कोई ऐसा गर्भ रहा है जिसका अपने आप गर्भपात हो गया था या आपने अपनी इच्छा से गर्भपात कराया था, या जिसका परिणाम एक मृत जन्म रहा हो?<br>**Have you ever had a pregnancy that miscarried, was aborted, or ended in a stillbirth?**	हाँ/Yes....................1<br>नहीं/No....................0<br>कोई जवाब नहीं/No response..........-99	009a=1
ABT 2	ऐसा गर्भ समापन आखरी बार कब हुआ?<br>**When did the last such pregnancy end?**<br>*अगर कोई प्रतिक्रिया नहीं तो Jan 2020 दर्ज करें।*<br>*Enter Jan 2020 for no response.*	**महीना**/Month [ ]<br>**महीना**/Year [ ]	ABT1 = 1
ABT 3	उस गर्भ का अपने आप गर्भपात हो गया था या आपने अपनी इच्छा से गर्भपात कराया था, या जिसका परिणाम एक मृत जन्म रहा था ?<br>**Did that pregnancy end in miscarriage, an abortion, or a stillbirth?**	अपने आप हुआ गर्भपात/Miscarriage.......1<br>इच्छा से करवाया गया गर्भपात/Abortion...2<br>मृत जन्म/Stillbirth....................3<br>नहीं जानती/Don't know..........-88<br>कोई जवाब नहीं/No response..........-99	ABT1 = 1

ABT 4	ऐसे सबसे अंतिम गर्भ की समाप्ति के समय आप कितने महीने की गर्भवती थीं?<br>**How many months pregnant were you when the last such pregnancy ended?**<br>अगर कोई जवाब नहीं, *-99* दर्ज करें।<br>*Enter -99 for no response.*	महीना/Months ☐	ABT1 = 1
ABT 5	आपने किस प्रकार का गर्भपात करवाया? क्या आप गर्भपात के लिए सुविधा केंद्र डी एन सी (दुसरे शब्दों में सफाई करवाई) कराने गए या केवल दवा लेकर या कुछ और करके गर्भपात करवाया?<br><br>**What type of abortion did you have? Did you go to a health facility for D&C (in other words "cleaning"), only take medication, or do something else?**	डी एन सी/"सफाई"/D&C/"cleaning" ....1<br>केवल दवा ली/Only took medication ....2<br>अन्य/Something else ....3<br>नहीं जानती/Don't know....................-88<br>कोई जवाब नहीं/No response ....-99	ABT3 = 2
ABT 6a	यह गर्भपात कहाँ किया गया था?<br>Where was the abortion performed?	**सार्वजनिक स्वास्थ्य के क्षेत्र/ Public Health Sector**<br>सरकार। / नगर अस्पताल/ Govt./Municipal Hospital .... 11<br>सरकार। औषधालय/ Govt. Dispensary .. 12<br>यूएफडब्ल्यूसी/यूएचसी/यूएचपी/UFWC/ UHC/UHP/UFWC .... 13<br>सीएचसी / ग्रामीण अस्पताल / ब्लॉक पीएचसी/ CHC/Rural Hospital/Block PHC .... 14<br>उप-केन्द्र / एएनएम/ Sub-Centre/ANM... 15<br>सरकारी मोबाइल क्लिनिक/ Govt. Mobile Clinic .... 16<br>कैम्प/ Camp .... 17<br>गैर सरकारी (NGO) संगठन या ट्रस्ट अस्पताल / क्लिनिक/ NGO or Trust Hospital/Clinic .... 21<br><br>**निजी (प्राइवेट) स्वास्थ्य क्षेत्र/ Private Health Sector**<br>प्राइवेट अस्पताल/ Pvt. Hospital .... 31<br>प्राइवेट चिकित्सक / क्लिनिक/ Pvt. Doctor/Clinic..................................... 32<br>प्राइवेट मोबाइल क्लिनिक/ Pvt. Mobile Clinic .... 33<br>प्रशिक्षित दाई के घर (टीबीए)/ Home of dai (TBA) .... 37<br><br>घर/ Home…………………………….51<br>अन्य/ Other ....96<br>जानती नहीं/ Don't know…………….-88<br>कोई जवाब नहीं/ No Response………………………... -99	ABT5 = 1 OR 3 OR -88

ABT 6b	जो गर्भपात के लिए आपने दवा ली वो आपने कहाँ से प्राप्त की?<br>Where did you obtain the abortion medicine?	**सार्वजनिक स्वास्थ्य के क्षेत्र/ Public Health Sector**<br>सरकार। / नगर अस्पताल/ Govt./Municipal Hospital .... 11<br>सरकार। औषधालय/ Govt. Dispensary .. 12<br>यूएफडब्ल्यूसी/यूएचसी/यूएचपी/UFWC/ UHC/UHP/UFWC .... 13<br>सीएचसी / ग्रामीण अस्पताल / ब्लॉक पीएचसी/ CHC/Rural Hospital/Block PHC .... 14<br>उप-केन्द्र / एएनएम/ Sub-Centre/ANM... 15<br>सरकारी मोबाइल क्लिनिक/ Govt. Mobile Clinic .... 16<br>कैम्प/ Camp .... 17<br>आंगनवाड़ी / आईसीडीएस केंद्र/ Anganwadi/ICDS Centre .... 18<br>आशा/ ASHA .... 19<br>अन्य समुदाय आधारित कार्यकर्ता/ Other Community-Based Worker .... 10<br>गैर सरकारी (NGO) संगठन या ट्रस्ट अस्पताल / क्लिनिक/ NGO or Trust Hospital/Clinic .... 21<br>**निजी (प्राइवेट) स्वास्थ्य क्षेत्र/ Private Health Sector**<br>प्राइवेट अस्पताल/ Pvt. Hospital .... 31<br>प्राइवेट चिकित्सक / क्लिनिक/ Pvt. Doctor/Clinic .... 32<br>प्राइवेट मोबाइल क्लिनिक/ Pvt. Mobile Clinic .... 33<br>वैद्य / हकीम / होमियोपैथ (आयुष)/ Vaidya/Hakim/Homeopath (Ayush) .. 34<br>पारंपरिक नीम हकीम / Traditional Healer 35<br>फार्मेसी / दवा की दुकान/ Pharmacy/Drugstore .... 36<br>दाई (टीबीए)/ Dai (TBA) .... 37<br>**अन्य स्रोत/ Other Source**<br>दुकान/ Shop .... 41<br>दोस्त / रिश्तेदार/ Friend/Relative .... 42<br>घर/ Home .... 51<br>अन्य/ Other .... 96<br>जानती नहीं/ Don't know .... -88<br>कोई जवाब नहीं/ No Response .... -99	ABT5 = 2
ABT 7	गर्भपात की दवाई, जो आपने ली, क्या आपके पास डॉक्टर द्वारा लिखा गया पर्चा था?<br>Did you have a prescription for the abortion medication?	हाँ/Yes .... 1<br>नहीं/No .... 0<br>नहीं जानती/Don't know .... -88<br>कोई जवाब नहीं/No response .... -99	ABT6b = 36 OR 41 OR 42 OR 51

ABT 8	गर्भपात किसके द्वारा किया गया?<br>**Who performed the abortion?**	डॉक्टर/Doctor....................................1<br>नर्स/ए.एन.एम/एल.एच.वी./Nurse/ANM/LHV ....2<br>दाई/Dai ....3<br>परिवार का सदस्य/रिश्तेदार/मित्र/Family member/relative/friend ....4<br>स्वयं/Self...5<br>अन्य/Other.......................................96<br>नहीं जानती/Don't know....................-88<br>कोई जवाब नहीं/No response............-99	ABT5 = 1 OR 3 OR -88
209a	आपकी पिछली माहवारी कब शुरू हुई?<br>**When did your last menstrual period start?**<br>*अगर आप दिन, सप्ताह, महीनों या वर्षों का चयन करते हैं, तो अगले स्क्रीन पर X की जगह एक नंबर दर्ज होगा।*<br>*आज के लिए 0 दिन दर्ज करें ना कि 0 सप्ताह / माह / वर्ष।*<br>*If you select days, weeks, months or years, you will enter a number for x on the next screen.*<br>*Enter 0 days for today, not 0 weeks/months/years.*	[ ] दिनों पहले/days ago<br>[ ] सप्ताहों पहले/weeks ago<br>[ ] महीनों पहले/months ago<br>[ ] वर्षों पहले/years ago<br>मासिक धर्म बंद / गर्भाशय आपरेशन से निकाल लिया गया है/Menopausal / Hysterectomy..................................5<br>पिछले जन्म से पूर्व/Before last birth ....6<br>कभी माहवारी नहीं हुई/Never menstruated....................................7<br>कोई जवाब नहीं/No response............-99	009a = 1
209b	कुछ महिलाएं गर्भाशय निकालने हेतु ऑपरेशन करवाती है। क्या आपने कोई ऐसा ऑपरेशन करवाया है?<br>**Some women undergo an operation to remove the uterus. Have you undergone such an operation?**	हाँ/Yes..1<br>नहीं/No ....0<br>अनिश्चित/Unsure ....2<br>कोई जवाब नहीं/No response............-99	209 ≠ 5 AND 209 > 6 months
210a	क्या आप अभी/वर्तमान में गर्भवती हैं ?<br>**Are you pregnant now?**	हाँ/Yes..1<br>नहीं/No ....0<br>अनिश्चित/Unsure ....2<br>कोई जवाब नहीं/No response............-99	209 ≠ 5
210b	*आप कितने महीने से गर्भवती हैं?*<br>हाल में दिया गया जन्म: [सबसे हाल ही में दिए गए जन्म की तारिख] को हुआ था<br>**How many months pregnant are you?**<br>**The most recent birth was: [Date of most recent birth]**<br>*दर्ज करें कितने माह पूरे हो चुके हैं. अगर कोई जवाब नहीं, -99 दर्ज करें, अगर पता नहीं, -88 दर्ज करें*<br>*Please record the number of completed months. Enter -88 for do not know, -99 for no response.*	**महीनों की संख्या**/ Number of months [ ]	210a = 1

211a	अब मैं भविष्य के बारे में कुछ सवाल पूछूंगी।<br>क्या आप कोई बच्चा/और बच्चा चाहेंगी या आप कोई बच्चा/ और बच्चे नहीं चाहेंगी ?<br>**Now I have some questions about the future. Would you like to have a/another child or would you prefer not to have any / any more children?**	कोई बच्चा/ और बच्चा चाहेंगी/Have a/another child ....1<br>कोई बच्चा/ और बच्चा नहीं चाहेंगी/No more/prefer no children...................2<br>वह कहती हैं गर्भवती नहीं हो सकतीं/Says she can't get pregnant ....3<br>अनिश्चित/ जानते नहीं/Undecided / Don't know ....-88<br>कोई जवाब नहीं/No response............-99	210a ≠ 1
211b	अब मैं भविष्य के बारे में कुछ सवाल पूछूंगी।<br>अभी होनेवाले बच्चे के बाद आप और बच्चा चाहेगीं, या क्या आप चाहेगीं और अधिक बच्चे न हों?<br>**Now I have some questions about the future. After the child you are expecting now, would you like to have another child, or would you prefer not to have any more children?**	और बच्चे चाहेंगी/Have a/another child ..1<br>और बच्चा नहीं चाहेंगी/No more/prefer no children ....2<br>वह कहती हैं गर्भवती नहीं हो सकतीं/Says she can't get pregnant ....3<br>अनिश्चित/ जानते नहीं/Undecided / Don't know ....-88<br>कोई जवाब नहीं/No response............-99	210a = 1
212a	आप एक/एक और बच्चे के लिए कितनी प्रतिक्षा करना चाहती हैं?<br>**How long would you like to wait from now before the birth of a/another child?**<br>*अगर आप महीनों या वर्षों का चयन करते हैंए तो अगली स्क्रीन पर ग् के जगह एक नंबर दर्ज करें।*<br>*अगर 36 महीनों से अधिक होंए श्सालश् का चयन करें।*<br>*जाँच लें कि आपने महीनों/वर्षों की उचित संख्या डाली हैं।*<br>*If you select months or years, you will enter a number for x on the next screen.*<br>*Select "Years" if more than 36 months.*<br>*Please check that you correctly entered the value for months/years.*	महीना/Months ☐<br>वर्ष/Years ☐<br>जल्द ही / अभी/Soon / now...................3<br>वह कहती हैं गर्भवती नहीं हो सकतीं/Says she can't get pregnant ....4<br>अन्य/Other ....5<br>अनिश्चित/जानती नहीं/Don't know ....-88<br>कोई जवाब नहीं/No response............-99	211a = 1

212b	अभी होनेवाले बच्चे के बाद आप एक और बच्चे के जन्म के लिए और कितनी प्रतिक्षा करना चाहेंगी?<br>**After the birth of the child you are expecting now, how long would you like to wait before the birth of another child?**<br>*अगर आप महीनों या वर्षों का चयन करते हैंए तो अगले स्क्रीन पर ग् के जगह एक नंबर दर्ज करें।*<br>*अगर 36 महीनों से अधिक होंए श्सालश् का चयन करें।*<br>*जाँच लें कि आपने महीनों/वर्षों की उचित संख्या डाली हैं।*<br>*If you select months or years, you will enter a number for x on the next screen.*<br>*Select "Years" if more than 36 months.*<br>*Please check that you correctly entered the value for months/years.*	महीना/Months [ ]<br>वर्ष/Years [ ]<br>जल्द ही / अभी/Soon / now....................3<br>वह कहती हैं गर्भवती नहीं हो सकतीं/Says she can't get pregnant ....4<br>अन्य/Other..5<br>अनिश्चित/जानती नहीं/Don't know.......-88<br>कोई जवाब नहीं/No response............-99	211b = 1
213a	अब मैं आपसे पिछले शिशु के जन्म के बारे में कुछ प्रश्न पूछना चाहती हूँ।<br>जिस समय आप गर्भवती हुईं, क्या आप गर्भवती होना चाहती थीं, या आप कुछ समय बाद तक प्रतीक्षा करना चाहती थीं या आप किसी/ और अधिक बच्चे को जन्म देना नहीं चाहती थीं ?<br>**Now I would like to ask a question about your last birth.**<br>**At the time you became pregnant, did you want to become pregnant then, did you want to wait until later, or did you not want to have any / any more children at all?**	तभी/Then..1<br>बाद में/Later ....2<br>बिल्कुल नहीं/Not at all..........................3<br>कोई जवाब नहीं/No response............-99	LCL_201 >0 AND 210a ≠ 1
213b	अब मैं आपकी वर्तमान गर्भावस्था के बारे में कुछ सवाल पूछना चाहती हूँ।<br>जिस समय आप गर्भवती हुईं, क्या आप गर्भवती होना चाहती थीं, या आप कुछ समय बाद तक प्रतीक्षा करना चाहती थीं या आप किसी बच्चे /और बच्चे को जन्म देना नहीं चाहती थीं ?<br>**Now I would like to ask a question about your current pregnancy.**<br>**At the time you became pregnant, did you want to become pregnant then, did you want to wait until later, or did you not want to have any / any more children at all?**	तभी/Then..1<br>बाद में/Later ....2<br>बिल्कुल नहीं/Not at all..........................3<br>कोई जवाब नहीं/No response............-99	210a = 1

**Section 3 – Contraception**<br>भाग 3 - गर्भनिरोधन<br>*अब मैं आपसे परिवार नियोजन के बारे में बात करना चाहती हूँ - ऐसे विभिन्न उपाय/विधियाँ जिनके प्रयोग से गर्भधारण को टाल/देरी कर सकते हैं।*<br>*कुछ तरीकों के लिए एक छवि स्क्रीन पर दिखाई देगी। यदि उत्तरदाता कहे कि उसने इस विधि के बारे में नहीं सुना है अथवा वह उत्तर देने में संकोच करे तो जोर से पढ़े और जोर देकर पूछें और यदि उपलब्ध हो तो छवि दिखाएें।*<br>*Now I would like to talk about family planning - the various ways or methods that a couple can use to delay or avoid a pregnancy.*<br>*An image will appear on the screen for some methods. If the respondent says that she has not heard of the method or if she hesitates to answer, read the probe aloud and show her the image, if available.*			
301a	क्या आपने कभी महिला नसबंदी के बारे में सुना है?<br>**Have you ever heard of female sterilization?**<br><br>विवरण: यह ओपरेशन महिला करवा सकती है जिससे और आगे बच्चे पैदा करने से रोका जा सकता है।<br>PROBE: Women can have an operation to avoid having any more children.<br><br>[NO IMAGE]	हाँ/Yes..........1<br>नहीं/No..........0<br>कोई जवाब नहीं/No response..........-99	009a = 1
301b	क्या आपने कभी पुरुष नसबंदी के बारे में सुना है?<br>**Have you ever heard of male sterilization?**<br><br>विवरण: यह ओपरेशन पुरूष करवा सकते है, जिससे और आगे बच्चे पैदा करने को रोका जा सकता है।<br>PROBE: Men can have an operation to avoid having any more children.<br><br>[NO IMAGE]	हाँ/Yes..........1<br>नहीं/No..........0<br>कोई जवाब नहीं/No response..........-99	009a = 1
301c	क्या आपने कभी गर्भनिरोधक इम्प्लांट लगवाने के बारे में सुना है?<br>**Have you ever heard of the contraceptive implant?**<br><br>विवरण: महिलायें डॉक्टर या नर्स से अपनी ऊपरी भुजा में एक या कई छोटे छड़ रख सकतें है जो एक या अधिक वर्षों के लिए गर्भ धारण को रोक सकतें है।<br>PROBE: Women can have one or several small rods placed in their upper arm by a doctor or nurse, which can prevent pregnancy for one or more years.<br><br>[IMAGE OF METHOD WILL APPEAR ON SCREEN]	हाँ/Yes..........1<br>नहीं/No..........0<br>कोई जवाब नहीं/No response..........-99	009a = 1
301d	क्या आपने कभी आईयूडी के बारे में सुना है?<br>**Have you ever heard of the IUD/PPIUD?**<br><br>विवरण: महिलाएं डॉक्टर या नर्स की मदद से गर्भाशय के अंदर T के आकार की कॉपर धातु की छड़ रख सकती है| PROBE: Women can have a loop or coil placed inside them by a doctor or a nurse.<br><br>[IMAGE OF METHOD WILL APPEAR ON SCREEN]	हाँ/Yes..........1<br>नहीं/No..........0<br>कोई जवाब नहीं/No response..........-99	009a = 1

301e	क्या आपने कभी गर्भनिरोधक इंजेक्शन (इन्जेक्टिबल्स) के बारे में सुना है?<br>**Have you ever heard of injectables?**<br><br>विवरण: महिलाएं स्वास्थ्य सेवा प्रदाता द्वारा एक इंजेक्शन लगवा सकतीं है जो एक या अधिक महीनों के लिए गर्भ धारण को रोक सकता है।<br>PROBE: Women can have an injection by a health provider that stops them from becoming pregnant for one or more months.<br><br>[IMAGE OF METHOD WILL APPEAR ON SCREEN]	हाँ/Yes..1<br>नहीं/No ....0<br>कोई जवाब नहीं/No response ....-99	009a = 1
301f	क्या आपने कभी (जन्म नियंत्रण) गोली के बारे में सुना है?<br>**Have you ever heard of the (birth control) pill?**<br><br>विवरण: महिलाएं गर्भवती होने से बचने के लिए हर दिन एक गोली ले सकती हैं।<br>PROBE: Women can take a pill every day to avoid becoming pregnant.<br><br>[IMAGE OF METHOD WILL APPEAR ON SCREEN]	हाँ/Yes..1<br>नहीं/No ....0<br>कोई जवाब नहीं/No response ....-99	009a = 1
301g	क्या आपने कभी आपातकालीन गर्भनिरोधक के बारे में सुना है?<br>**Have you ever heard of emergency contraception?**<br><br>विवरण: असुरक्षित संभोग के बाद गर्भावस्था को रोकने के लिए महिलाऐं एक आपातकालीन उपाय के रूप में ये विशेष गोलियाँ 5 दिनों के भीतर किसी भी समय ले सकती हैं।<br>PROBE: As an emergency measure after unprotected sexual intercourse women can take special pills at any time within five days to prevent pregnancy.<br><br>[NO IMAGE]	हाँ/Yes..1<br>नहीं/No ....0<br>कोई जवाब नहीं/No response ....-99	009a = 1
301h	क्या आपने कभी कंडोम के बारे में सुना है?<br>**Have you ever heard of male condoms/Nirodh?**<br><br>विवरण: पुरुष संभोग से पहले अपने लिंग पर एक पतली झिल्ली चढ़ा सकते हैं।<br>PROBE: Men can put a rubber sheath on their penis before sexual intercourse.<br><br>[IMAGE OF METHOD WILL APPEAR ON SCREEN]	हाँ/Yes..1<br>नहीं/No ....0<br>कोई जवाब नहीं/No response ....-99	009a = 1

301i	क्या आपने कभी महिला कंडोम के बारे में सुना है?<br>**Have you ever heard of female condoms?**<br><br>विवरण: महिलाऐं संभोग से पहले उनकी योनी में एक झिल्ली डाल सकती हैं।<br>PROBE: Women can put a sheath in their vagina before sexual intercourse.<br><br>[IMAGE OF METHOD WILL APPEAR ON SCREEN]	हाँ/Yes..1<br>नहीं/No...0<br>कोई जवाब नहीं/No response............-99	009a = 1
301j	क्या आपने कभी मानक दिवस विधि या साईकिल बीड्स के बारे में सुना है ?<br>**Have you ever heard of the standard days method or Cycle Beads?**<br><br>विवरण:<br>एक औरत कौन से दिनों में गर्भवती हो सकती है, यह पता करने के लिए एक रंगीन मोतियों की माला का उपयोग किया जा सकता है। जिन दिनों में वह गर्भधारण कर सकती है उन दिनों में संभोग के दौरान कंडोम का इस्तेमाल करें या संभोग ना करें।<br>PROBE: A Woman can use a string of colored beads to know the days she can get pregnant. On the days she can get pregnant, she and her partner use a condom or do not have sexual intercourse.<br><br>[IMAGE OF METHOD WILL APPEAR ON SCREEN]	हाँ/Yes..1<br>नहीं/No...0<br>कोई जवाब नहीं/No response............-99	009a = 1
301k	क्या आपने कभी स्तनपान अन्तराल विधि (LAM) के बारे में सुना है?<br>**Have you ever heard of the Lactational Amenorrhea Method or LAM?**<br><br>[NO DESCRIPTION; NO IMAGE]	हाँ/Yes..1<br>नहीं/No...0<br>कोई जवाब नहीं/No response............-99	009a = 1
301l	क्या आपने कभी लय विधि (रिदम मेथड) के बारे में सुना है?<br>**Have you ever heard of the rhythm method?**<br><br>विवरण:जिन दिनों में गर्भ धारण हो सकता है उनमें संभोग नहीं करके महिला गर्भधारण से बच सकती है।<br>PROBE: Women can avoid pregnancy by not having sexual intercourse on the days of the month they think they can get pregnant.<br><br>[NO IMAGE]	हाँ/Yes..1<br>नहीं/No...0<br>कोई जवाब नहीं/No response............-99	009a = 1

301 m	क्या आपने कभी बाह्य स्खलन (विड्रावल) विधि के बारे में सुना है ?<br>**Have you ever heard of the withdrawal method?**<br><br>विवरण: पुरूष सावधान रह सकते हैं और चरमोत्कर्ष के समय बाहर निकाल सकते हैं<br>PROBE: Men can be careful and pull out before climax.<br><br>[NO IMAGE]	हाँ/Yes....1<br>नहीं/No....0<br>कोई जवाब नहीं/No response....-99	009a = 1
301n	क्या आपने कभी किन्हीं अन्य विधियों के बारे में सुना है, महिला या पुरूष जिनका उपयोग करके गर्भधारण टाल सकते हैं?<br>**Have you ever heard of any other ways or methods that women or men can use to avoid pregnancy?**	हाँ/Yes....1<br>नहीं/No....0<br>कोई जवाब नहीं/No response....-99	009a = 1
302a	क्या आप या आपके साथी वर्तमान में गर्भवती होने से टालने के लिए या देरी करने के लिए कुछ कर रहे हैं या किसी भी विधि का उपयोग कर रहे हैं ?<br>**Are you or your partner currently doing something or using any method to delay or avoid getting pregnant?**	हाँ/Yes....1<br>नहीं/No....0<br>कोई जवाब नहीं/No response....-99	210a ≠ 1 AND 009a=1
302b	आप कौन सी विधि या विधियों का उपयोग कर रहे हैं?<br>**Which method or methods are you using?**<br><br>पूछें: कोई और?<br>**Probe: Anything else?**<br><br>*उल्लिखित सभी तरीकों का चयन करें। सभी विकल्प देखने के लिए नीचे स्क्रॉल करें।*<br>*Select all methods mentioned. Scroll to the bottom to see all choices.*	महिला नसबंदी/Female sterilization ..1/0<br>पुरुष नसबंदी/Male sterilization ....1/0<br>छड़(इम्प्लांट)/Implant....1/0<br>आईयूडी / पिपिआईयूडी/IUD/PPIUD ...1/0<br>इंजेक्शन /Injectables....1/0<br>गोली/Pill....1/0<br>आपातकालीन गर्भनिरोधक/Emergency Contraception ....1/0<br>पुरुष कंडोम / निरोध/Male Condom/Nirodh....1/0<br>महिला कंडोम/Female Condom ....1/0<br>मानकदिन/ साइकिल बीड्स /Std. Days/Cycle beads....1/0<br>लैम/LAM....1/0<br>रिदम मेथड/Rhythm method....1/0<br>बाह्य स्खलन(विद्ड्रावल)/Withdrawal....1/0<br>अन्य पारंपरिक तरीके/Other traditional methods....1/0<br>कोई जवाब नहीं/No response…….-99	302a = 1
LCL3 01	क्या आपने कभी नसबंदी करवायी है?<br>Have you ever been sterilized?	हाँ/Yes....1<br>नहीं/No....0<br>कोई जवाब नहीं/No response....-99	302a = 0 OR 302b ≠ female sterilization

CALC CM	CALCULATE: CURRENT METHOD<br>THIS WILL NOT APPEAR ON THE SCREEN<br>ODK will identify the most effective method currently being used by the respondent by selecting the highest method in the choice list	महिला नसबंदी/Female Sterilization.....1<br>पुरुष नसबंदी/Male Sterilization...........2<br>छड़(इम्प्लांट)/Implant..........................3<br>आईयूडी / पिपिआईयूडी/IUD/PPIUD ......4<br>इंजेक्शन /Injectables..........................5<br>गोली/Pill ....7<br>आपातकालीन गर्भनिरोधक/Emergency Contraception ....8<br>पुरुष कंडोम / निरोध/Male Condom/Nirodh...............................9<br>महिला कंडोम/Female Condom ....10<br>मानकदिन/ साइकिल बीड्स /Std. Days/Cycle beads..........................13<br>लैम/LAM ....14<br>रिदम मेथड/Rhythm method..............30<br>बाह्य स्खलन(विद्ड्रावल)/Withdrawal.....31<br>अन्य पारंपरिक तरीके/Other traditional methods ....39<br>कोई जवाब नहीं/No response………………………....-99	302a=1 AND 302b ≠-99 OR LCL301 = 1
302c	क्या आप गर्भवती होने में देरी करने के लिए स्तनपान करवा रही हैं ?<br>**Are you breastfeeding to delay or avoid becoming pregnant?**	हाँ/Yes..1<br>नहीं/No ....0<br>कोई जवाब नहीं/No response............-99	302b = LAM
303	क्या आपके प्रदाता ने आपको या आपके साथी को बताया था कि यह विधि स्थायी है?<br>**Did the provider tell you or your partner that this method was permanent?**	हाँ/Yes..1<br>नहीं/No ....0<br>कोई जवाब नहीं/No response............-99	302b = male or female sterilization OR LCL301 = 1
304	क्या आप ऐसी किसी जगह को जानती हैं जहाँ से आप परिवार नियोजन के साधन प्राप्त कर सकती हैं ?<br>**Do you know of a place where you can obtain a method of family planning?**	हाँ/Yes..1<br>नहीं/No ....0<br>कोई जवाब नहीं/No response............-99	302a ≠ 1 OR LCL301 ≠ 1
305a	आपने कहा कि वर्तमान में आप गर्भनिरोधक विधि का उपयोग नहीं कर रहे हैं। क्या आप सोचते हैं कि भविष्य में किसी भी समय गर्भवती होने से बचने के लिए या देरी करने के लिए गर्भ निरोधक विधि का उपयोग करेंगे ?<br>**You said that you are not currently using a contraceptive method. Do you think you will use a contraceptive method to delay or avoid getting pregnant at any time in the future?**	हाँ/Yes..1<br>नहीं/No ....0<br>कोई जवाब नहीं/No response............-99	302a ≠ 1 AND 210a ≠ 1<br>AND LCL301 ≠ 1

305b	क्या आप भविष्य में किसी भी समय गर्भवती होने से टालने के लिए या देरी करने के लिए गर्भनिरोधक विधि का उपयोग करेगें?<br>**Do you think you will use a contraceptive method to delay or avoid getting pregnant at any time in the future?**	हाँ/Yes..1<br>नहीं/No..0<br>कोई जवाब नहीं/No response...........-99	302a ≠ 1 AND 210a=1 AND LCL301 ≠ 1
306a	पिछले 12 महीनों में, क्या आपने गर्भवती होने से टालने के लिए या देरी करने के लिए कुछ किया या गर्भनिरोधक विधि का उपयोग किया?<br>**In the last 12 months, have you ever done something or used a method to delay or avoid getting pregnant?**	हाँ/Yes..1<br>नहीं/No..0<br>कोई जवाब नहीं/No response...........-99	302a ≠ 1 AND LCL301 ≠ 1
306b	आपने हाल ही में कौन सी विधी अपनायी?<br>**Which method did you use most recently?**<br><br>पूछें: और कुछ?<br>**Probe: Anything else?**<br><br>*सबसे प्रभावी तरीके का चयन करें (सूची में से सबसे उपर की विधि)। सभी विकल्प देखने के लिए नीचे स्क्राल करें।*<br>*Select most effective method (highest method on list). Scroll to bottom to see all choices.*	छड़(इम्प्लांट)/Implant..........................3<br>आईयूडी / पिपिआईयूडी/IUD/PPIUD ......4<br>इंजेक्शन/Injectables...........................5<br>गोली/Pill..7<br>आपातकालीन गर्भनिरोधक/Emergency Contraception ....8<br>पुरुष कंडोम / निरोध/Male Condom/Nirodh...............................9<br>महिला कंडोम/Female Condom ....10<br>मानकदिन/ साइकिल बीड्स /Std. Days/Cycle beads.........................13<br>लैम/LAM ....14<br>रिदम मेथड/Rhythm method.............30<br>बाह्य स्खलन(विद्ड्रावल)/Withdrawal.....31<br>अन्य पारंपरिक तरीके/Other traditional methods..39<br>कोई जवाब नहीं/No response………………………...-99	306a = 1
307	जब आपने **[हाल ही कि/वर्तमान मे प्रयोग ली जा रही]** विधि का प्रयोग करना आरम्भ किया तो क्या आपने अपने पति/साथी के साथ गर्भ को रोकने के लिए या देरी करने के बारे में चर्चा की थी।<br>**Before you started using [MOST RECENT / CURRENT METHOD], had you discussed the decision to delay or avoid pregnancy with your husband/partner?**	हाँ/Yes..1<br>नहीं/No..0<br>नहीं जानती/Don't know...................-88<br>कोई जवाब नहीं/No response -99	302a = 1 OR 306a = 1 OR LCL301 = 1
308	गर्भनिरोधक विधि का प्रयोग करना मुख्य रूप से आपका निर्णय था, या मुख्य रूप से आपके पति/साथी का या आप दोनों का संयुक्त निर्णय था?<br>**Would you say that using contraception is mainly your decision, mainly your husband/partner's decision or did you both decide together?**	मुख्यतः उत्तरदाता का/Mainly respondent1<br>मुख्यतः/Mainly husband/partner.........2<br>सयुंक्त निर्णय था/Joint Decision............3<br>अन्य/Other......................................96<br>कोई जवाब नहीं/No response...........-99	302a = 1 OR 306a = 1 OR LCL301 = 1

309a	किस महीने एवं वर्ष से आप बिना रूके [वर्तमान मे प्रयोग ले रही] विधि का प्रयोग कर रहीं हैं।<br>**Since what month and year have you been using [CURRENT METHOD] without stopping?**<br>*यदि आवश्यक हो तो किन्हीं यादगार घटनाओं से आगे या पीछे की गणना की जानी चाहिए।*<br>*Calculate backwards from memorable events if needed.*<br><br>हाल में दिया गया जन्म: [mm-yyyy]<br>वर्तमान शादी: [mm-yyyy]<br>**Most Recent Birth: [mm-yyyy]**<br>**Current Marriage: [mm-yyyy]**<br><br>*आज से पहले होना चाहिए। उत्तरदाता कम से कम 10 साल का होना चाहिए।*<br>*अगर कोई जवाब नहीं, Jan 2020 दर्ज करें।*<br>*Must be before today. Respondent must be at least 10 years old.*<br>*Enter Jan 2020 for no response.*	**महीना**/Month ☐<br>**साल**/Year ☐	302a=1 OR LCL301 = 1
309b	आपने [MOST RECENT METHOD] का प्रयोग कब बन्द किया ?<br>**When did you stop using your [MOST RECENT METHOD]?**<br>*तारीख को दर्ज करें।*<br>*तारीख जानने के लिए यदि जरूरत हो तो यादगार घटनाओं के आधार पर पीछे की उम्र की गणना की जानी चाहिए।*<br>*अगर कोई जवाब नहीं, Jan 2020 दर्ज करें।*<br>*Please record the date.*<br>*The date should be found by calculating backwards from memorable events if needed.*<br>*Enter Jan 2020 for no response.*	**महीना**/Month ☐<br>**साल**/Year ☐	306a=1

309c	प्रयोग बंद करने से पूर्व किस महीने एवं वर्ष से आपने [MOST RECENT METHOD] का प्रयोग करना आरम्भ किया?<br><br>In what month and year had you started using [MOST RECENT METHOD] before stopping?<br><br>*यदि आवश्यक हो तो किन्हीं यादगार घटनाओं से आगे या पीछे की गणना की जानी चाहिए।*<br>*Calculate backwards from memorable events if needed.*<br><br>हाल में दिया गया जन्म: [mm-yyyy]<br>वर्तमान शादी: [mm-yyyy]<br>Most Recent Birth: [mm-yyyy]<br>Current Marriage: [mm-yyyy]<br><br>*आज से पहले होना चाहिए। उत्तरदाता कम से कम 10 साल का होना चाहिए।*<br>*अगर कोई जवाब नहीं, Jan 2020 दर्ज करें।*<br>*Must be before today. Respondent must be at least 10 years old.*<br><br>*Enter Jan 2020 for no response.*	**महीना**/Month [ ]<br>**साल**/Year [ ]	306a=1
309d	जाँचेंः मैं सुनिश्चित करना चाहती हूँ कि मैं सहीं हूँ: आपने बिना रुकावट के [RECENT METHOD] का प्रयोग लगातार [START DATE] से [END DATE] तक किया है। क्या मै सही हूँ?<br>CHECK: Just to make sure I have this correct, you used [RECENT METHOD] continuously between [START DATE] and [END DATE] without stopping, is that correct?	हाँ/Yes...1<br>नहीं/No 0	306a=1
	पिछली स्क्रीन पर जाएं और सबसे हाल ही में प्रयोग ली गयी विधि की अवधि सुनिश्चित करने हेतु गहराई से जाँचें।<br><br>GO BACK TO THE PREVIOUS SCREEN AND PROBE TO DETERMINE THE PERIOD OF MOST RECENT CONTINUOUS USE.<br><br>सुझावित जाँचः<br>- अंतिम बार आपने *[METHOD]* का प्रयोग कब किया था?<br>- आपने *[METHOD]* का प्रयोग बिना रुके कितने समय तक किया?<br><br>*Suggested probes:*<br>- *When was the last time you used [METHOD]?*<br>- *How long had you been using [METHOD] without stopping*		309d = 0

310	आपने क्यों [MOST RECENT METHOD] का उपयोग बंद किया?<br><br>**Why did you stop using [MOST RECENT METHOD]?**	अनियमित सेक्स/ पति दूर/Infrequent sex / husband away.................................1<br>उपयोग करते समय गर्भधारण/Became pregnant while using........................2<br>गर्भवती होना चाहती थी/Wanted to become pregnant ....3<br>पति/साथी की अस्वीकृति/Husband / partner disapproved.........................4<br>ज्यादा प्रभावी तरीका चाहती थी/Wanted more effective method ....5<br>कोई विधि उपलब्ध नहीं/No method available...6<br>स्वास्थ्य संबंधी समस्याएं/Health concerns7<br>दुष्प्रभाव का डर/Fear of side effects.....8<br>पहुंच से बाहर/ बहुत दूर/Lack of access / too far..9<br>बहुत अधिक क़ीमत/Costs too much ....10<br>उपयोग में असुविधा/Inconvenient to use ....11<br>भाग्यवादी/Fatalistic ....12<br>गर्भधारण करना कठिन / मासिक धर्म बंद/Difficult to get pregnant / menopausal ....13<br>शारीरिक प्रक्रियाओं में हस्तक्षेप/Interferes with body's processes....................14<br>अन्य/Other.......................................96<br>नहीं जानती/Don't know....................-88<br>कोई जवाब नहीं/No response............-99	306a=1

311a	आपने [CURRENT/MOST RECENT METHOD] का उपयोग [DATE FROM 310a or 310c] से शुरू किया। आपने या आपके साथी ने इसेउस समय कहाँ से प्राप्त किया? यूएफड्ब्ल्युसी<br>**You first started using [CURRENT/MOST RECENT METHOD] in [DATE FROM 310a or 310c]. Where did you or your partner get it at that time?**<br><br>*सभी विकल्प देखने के लिए नीचे स्क्रॉल करें।*<br>*Scroll to bottom to see all choices.*	**सार्वजनिक स्वास्थ्य के क्षेत्र/ Public Health Sector**<br>सरकार। / नगर अस्पताल/ Govt./Municipal Hospital .... 11<br>सरकार। औषधालय/ Govt. Dispensary .. 12<br>यूएफडब्ल्यूसी/यूएचसी/यूएचपी/UFWC/ UHC/UHP .... 13<br>सीएचसी / ग्रामीण अस्पताल / ब्लॉक पीएचसी/ CHC/Rural Hospital/Block PHC .... 14<br>उप-केन्द्र / एएनएम/ Sub-Centre/ANM... 15<br>सरकारी मोबाइल क्लिनिक/ Govt. Mobile Clinic .... 16<br>कैम्प/ Camp .... 17<br>आंगनवाड़ी / आईसीडीएस केंद्र/ Anganwadi/ICDS Centre .... 18<br>आशा/ ASHA .... 19<br>अन्य समुदाय आधारित कार्यकर्ता/ Other Community-Based Worker .... 10<br>गैर सरकारी (NGO) संगठन या ट्रस्ट अस्पताल / क्लिनिक/ NGO or Trust Hospital/Clinic .... 21<br><br>**निजी (प्राइवेट) स्वास्थ्य क्षेत्र/ Private Health Sector**<br>प्राइवेट अस्पताल/ Pvt. Hospital .... 31<br>प्राइवेट चिकित्सक / क्लिनिक/ Pvt. Doctor/Clinic .... 32<br>प्राइवेट मोबाइल क्लिनिक/ Pvt. Mobile Clinic .... 33<br>वैद्य / हकीम / होमियोपैथ (आयुष)/ Vaidya/Hakim/Homeopath (Ayush) .. 34<br>पारंपरिक नीम हकीम / Traditional Healer 35<br>फार्मेसी / दवा की दुकान/ Pharmacy/Drugstore .... 36<br>दाई (टीबीए)/ Dai (TBA) .... 37<br>**अन्य स्रोत/ Other Source**<br>दुकान/ Shop .... 41<br>दोस्त / रिश्तेदार/ Friend/Parent/Relative .... 42<br><br>अन्य/ Other .... 96<br>जानती नहीं/ Don't know.......... -88<br>कोई जवाब नहीं/ No Response.......... -99	(CALC_CM ≠14, 30, 31, 39, -99)<br><br>OR<br><br>(306b ≠ 14, 30, 31, 39, -99)

311b	आपने **[RHYTHM/LACTATIONAL AMENORRHEA METHOD]** का प्रयोग कर्णं कहाँ/किससे सीखा?<br>**Where did you learn how to use [RHYTHM/LACTATIONAL AMENORRHEA METHOD]?**<br><br>*सभी विकल्प देखने के लिए नीचे स्क्रॉल करें।*<br>*Scroll to bottom to see all choices.*	**सार्वजनिक स्वास्थ्य के क्षेत्र/ Public Health Sector**<br>सरकार। / नगर अस्पताल/ Govt./Municipal Hospital .... 11<br>सरकार। औषधालय/ Govt. Dispensary .. 12<br>यूएफडब्ल्यूसी/यूएचसी/यूएचपी/UFWC/ UHC/UHP .... 13<br>सीएचसी / ग्रामीण अस्पताल / ब्लॉक पीएचसी/ CHC/Rural Hospital/Block PHC .... 14<br>उप-केन्द्र / एएनएम/ Sub-Centre/ANM... 15<br>सरकारी मोबाइल क्लिनिक/ Govt. Mobile Clinic .... 16<br>कैम्प/ Camp .... 17<br>आंगनवाड़ी / आईसीडीएस केंद्र/ Anganwadi/ICDS Centre .... 18<br>आशा/ ASHA...................................... 19<br>अन्य समुदाय आधारित कार्यकर्ता/ Other Community-Based Worker .... 10<br>गैर सरकारी (NGO) संगठन या ट्रस्ट अस्पताल / क्लिनिक/ NGO or Trust Hospital/Clinic .... 21<br><br>**निजी (प्राइवेट) स्वास्थ्य क्षेत्र/Private Health Sector**<br>प्राइवेट अस्पताल/ Pvt. Hospital .... 31<br>प्राइवेट चिकित्सक / क्लिनिक/ Pvt. Doctor/Clinic...................................... 32<br>प्राइवेट मोबाइल क्लिनिक/ Pvt. Mobile Clinic .... 33<br>वैद्य / हकीम / होमियोपैथ (आयुष)/ Vaidya/Hakim/Homeopath (Ayush) .. 34<br>पारंपरिक नीम हकीम / Traditional Healer 35<br>फार्मेसी / दवा की दुकान/ Pharmacy/Drugstore .... 36<br>दाई (टीबीए)/ Dai (TBA) .... 37<br>**अन्य स्रोत/Other Source**<br>दुकान/ Shop....................................... 41<br>दोस्त / रिश्तेदार/ Friend/Parent/Relative .... 42<br><br>अन्य/ Other ....96<br>जानती नहीं/ Don't know…………….-88<br>कोई जवाब नहीं/ No Response……………………….... -99	(CALC_CM =14, or 30)<br><br>OR<br><br>306b = 14 or 30)

312a	आपने जब [MOST RECENT / CURRENT METHOD] को प्राप्त किया तो गर्भधारण टालने के लिए या देरी करने के लिए उस विधि से होने वाले दुष्प्रभाव या समस्याओं के बारे में क्या प्रदाता ने आपको बताया था?<br>**When you obtained your [MOST RECENT / CURRENT METHOD], were you told by the provider about side effects or problems you might have with a method to delay or avoid pregnancy?**	हाँ/Yes..1<br>नहीं/No..0<br>कोई जवाब नहीं/No response............-99	311a ≠ .
312b	क्या आपको बताया गया था कि दुष्प्रभाव या समस्याओं की स्थिति में क्या करना है?<br>**Were you told what to do if you experienced side effects or problems?**	हाँ/Yes..1<br>नहीं/No..0<br>कोई जवाब नहीं/No response............-99	312a = 1
313	उस समय परिवार नियोजन प्रदाता द्वारा क्या यह बताया गया था कि आप [MOST RECENT / CURRENT METHOD] के अलावा अन्य परिवार नियोजन के तरीकों का भी प्रयोग कर सकते है?<br>**At that time, were you told by a family planning provider about methods of family planning other than [MOST RECENT/CURRENT METHOD] that you could use?**	हाँ/Yes..1<br>नहीं/No..0<br>कोई जवाब नहीं/No response............-99	311a ≠ . OR 311b ≠ .
314a	जब आप गए तब क्या आपको वही विधि मिल गई जो आप चाहते थे ?<br>**During that visit, did you obtain the method you wanted to delay or avoid getting pregnant?**	हाँ/Yes..1<br>नहीं/No..0<br>कोई जवाब नहीं/No response............-99	311a ≠ .
314b	जब आपने **[LAM/Rhythm]** विधि का प्रयोग करना आरम्भ किया, क्या यह वही विधि थी जिसका उपयोग आप गर्भ टालने के लिए या स्थगित करने हेतु करना चाहते थे।<br>**When you began using [LAM/Rhythm] was this the method you wanted to use to delay or avoid getting pregnant?**	हाँ/Yes..1<br>नहीं/No..0<br>कोई जवाब नहीं/No response............-99	311b ≠ .

314c	जो विधि आप चाहते थे वही विधि आपने क्यों प्राप्त नहीं की ?<br>**Why didn't you obtain the method you wanted?**	उस दिन विधि स्टाक में नहीं थी/Method out of stock that day................................1<br>विधि कभी भी उपलब्ध नहीं थी/Method not available at all ....2<br>प्रदाता विधि प्रदान करने के लिए प्रशिक्षित नहीं था/Provider not trained to provide the method ....3<br>प्रदाता ने एक अलग विधि की सिफारिश की/Provider recommended a different method ....4<br>विधि को प्रयोग करने योग्य नहीं/Not eligible for method.......................................5<br>एक विधि को नहीं अपनाने का फैसला/Decided not to adopt a method6<br>बहुत महंगा/Too costly.........................7<br>अन्य/Other......................................96<br>जानती नहीं/Don't know....................-88<br>कोई जवाब नहीं/No response............-99	314a = 0
315a	इस विजिट के दौरान, विधि चुनने का अंतिम फैसला किसका था?<br>**During that visit, who made the final decision about what method you got?**	आपका स्वयं का/You alone ....1<br>प्रदाता का/Provider..............................2<br>साथी (सहयोगी) का/Partner ....3<br>आपका और प्रदाता का/You and provider4<br>आप और साथी (सहयोगी) का/You and partner ....5<br>अन्य/Other......................................96<br>जानती नहीं/Don't know....................-88<br>कोई जवाब नहीं/No response............-99	311a ≠ .
315b	**[LAM/Rhythm]** विधि प्रयोग करने का अंतिम निर्णय किसने लिया था?<br>**Who made the final decision to use [LAM/Rhythm]?**	आपका स्वयं का/You alone ....1<br>प्रदाता का/Provider..............................2<br>साथी (सहयोगी) का/Partner ....3<br>आपका और प्रदाता का/You and provider4<br>आप और साथी (सहयोगी) का/You and partner ....5<br>अन्य/Other......................................96<br>जानती नहीं/Don't know....................-88<br>कोई जवाब नहीं/No response............-99	311b ≠ .
316	क्या आप इस प्रदाता के पास वापस जायेंगे ?<br>**Would you return to this provider?**<br><br>प्रदाता: [Type of Provider from Q 311a]<br>**Provider: [Type of Provider from Q 311a]**	हाँ/Yes...1<br>नहीं/No ....0<br>नहीं जानती/Don't know....................-88<br>कोई जवाब नहीं/No response............-99	311a ≠ . , 42, AND 96
317	क्या आप अपने रिश्तेदार या दोस्त को इस प्रदाता/ सेवा केंद्र की सिफारिश करेगें?<br>**Would you refer your relative or friend to this provider / facility?**	हाँ/Yes...1<br>नहीं/No ....0<br>नहीं जानती/Don't know....................-88<br>कोई जवाब नहीं/No response............-99	311a ≠ . , 42, AND 96

318a	पिछले 12 महीनों में, आपने परिवार नियोजन सेवाओं (सबसे वर्तमान विधि सहित) के लिए कोई भी शुल्क दिया है?<br>**In the last 12 months, have you paid any fees for family planning services (including the most current method)?**	हाँ/Yes....1<br>नहीं/No....0<br>कोई जवाब नहीं/No response....-99	302a or 306a = 1 OR LCL301 = 1
318b	आपने कितना शुल्क दिया था?<br>**How much did you pay?**<br>*सभी कीमतों को रुपयों में दर्ज करें। अगर पता नहीं, -88 दर्ज करें, अगर कोई जवाब नहीं, -99 दर्ज करें।*<br>*Enter all prices in Rupees. Enter -88 if respondent does not know, -99 for no response.*	**शुल्क**/Fee ☐	318a = 1
319	क्या आपने कभी भी गर्भधारण नहीं करने/टालने के लिए किसी भी प्रकार की कोशिश की थी ?<br>**Have you ever done anything or tried in any way to delay or avoid getting pregnant?**	हाँ/Yes....1<br>नहीं/No....0<br>कोई जवाब नहीं/No response....-99	306a ≠ 1 OR 302a ≠ 1 OR LCL301 ≠ 1
320	आपकी उम्र कितनी थी जब आपने पहली बार गर्भावस्था को टालने के लिए या देरी करने के लिए किसी विधि का इस्तेमाल किया?<br>उत्तरदाता पिछले जन्मदिन पर [age from 102] साल की थी।<br>**How old were you when you first used a method to delay or avoid getting pregnant?**<br>**The respondent said she was [age from 102] years old at her last birthday.**<br>उम्र वर्षों में दर्ज करें।<br>अगर उत्तरदाता को पता नहीं, *-88* दर्ज करें।<br>अगर कोई जवाब नहीं, *-99* दर्ज करें।<br>उम्र *11* वर्ष से छोटी नहीं हो सकती।<br>*Enter the age in years.*<br>*Enter -88 if respondent does not know.*<br>*Enter -99 if there is no response.*<br>*Cannot be younger than 9.*	**उम्र**/Age ☐	302a=1 OR 306a=1 OR 319=1 OR LCL301 = 1
321	उस समय आपके कितने जीवित बच्चें थे?<br>नोट: उत्तरदाता कहतीं हैं [204 में] उसने [number of birth events] बार जन्म दिया।<br>**How many living children did you have at that time, if any?**<br>**Note: the respondent said that she gave birth [number of live births] times in 204.**<br>अगर कोई जवाब नहीं, *-99* दर्ज करें।<br>*Enter -99 for no response*	**संख्या**/Number ☐	Age in 320>0 AND 200=1

322	आपने पहली बार गर्भावस्था को टालने अथवा रोकने के लिए कौन सा तरीका इस्तेमाल किया था। ?<br>**Which method did you first use to delay or avoid getting pregnant?**<br><br>*विधि विकल्प नहीं पढ़ियें । सभी विकल्प देखने के लिए नीचे स्क्रॉल करें।*<br>*Do not read the method choices. Scroll to the bottom to see all choices.*	महिला नसबंदी/Female Sterilization.....1<br>पुरुष नसबंदी/Male Sterilization...........2<br>छड़(इम्प्लांट)/Implant...........................3<br>आईयूडी / पिपिआईयूडी/IUD/PPIUD ......4<br>इंजेक्शन /Injectables...........................5<br>गोली/Pill ....7<br>आपातकालीन गर्भनिरोधक/Emergency Contraception ....8<br>पुरुष कंडोम / निरोध/Male Condom/Nirodh................................9<br>महिला कंडोम/Female Condom ....10<br>मानकदिन/ साइकिल बीड्स /Std. Days/Cycle beads..........................13<br>लैम/LAM..14<br>रिदम मेथड/Rhythm method..............30<br>बाह्य स्खलन(विद्ड्रावल)/Withdrawal.....31<br>अन्य पारंपरिक तरीके/Other traditional methods ....39<br>कोई जवाब नहीं/No response…………………………....-99	319 = 1

323a	आपने कहा कि आपको कोई/और बच्चा नहीं चाहिए व आप गर्भावस्था को रोकने के लिए कोई परिवार नियोजन की विधि का उपयोग भी नहीं कर रही है<br>क्या आप हमें इसका मुख्य कारण बता सकती हैं कि गर्भावस्था को रोकने के लिए आप किसी विधि का उपयोग क्यों नहीं कर रहीं हैं। गहराई से पूछें: कोई भी अन्य कारण है?<br>**You said that you do not want any / anymore children and that you are not using a method to avoid pregnancy.**<br>**Can you tell me the reason why you are not using a method to prevent pregnancy?**<br>**PROBE: Any other reason?**<br>उल्लिखित सभी कारणों का चयन करें।<br>"जानती नहीं" या "कोई जवाब नहीं" अन्य विकल्पों के साथ चयन नहीं कर सकते।<br>अगर 104 का उत्तर "हाँ, वर्तमान में शादीशुदा है" तो " शादीशुदा नहीं" चयन नहीं कर सकते।<br>सभी विकल्प देखने के लिए नीचे स्क्रॉल करें।<br>*RECORD ALL REASONS MENTIONED.*<br>*Cannot select "Not married" if 104 is "Yes, currently married".*<br>*Scroll to the bottom to see all choices.*	शादीशुदा नहीं/Not married................1/0<br>कम बार सेक्स/ पति दूर/Infrequent sex / not having sex...............................1/0<br>रजोनिवृत्त/गर्भाशय निकलवा दिया /Menopausal/Hysterectomy ....1/0<br>उपजाऊ नहीं/Subfecund / infecund ..1/0<br>पिछले जन्म के बाद से माहवारी नहीं/Not menstruated since last birth..........1/0<br>स्तनपान/Breastfeeding....................1/0<br>पति कई दिनों से दूर ह/Husband away for multiple days................................1/0<br>भगवान के ऊपर / भाग्यवादी//Up to God / fatalistic...1/0<br>उत्तरदाता ने विरोध किया/Respondent opposed ....1/0<br>पति / पार्टनर ने विरोध किया/Husband / partner opposed............................1/0<br>दूसरों ने विरोध किया/Others opposed 1/0<br>धार्मिक निषेध/Religious prohibition ..1/0<br>कोई विधि नहीं जानती/Knows no method ....1/0<br>कोई स्रोत नहीं जानती/Knows no source ....1/0<br>दुष्प्रभाव का डर/Fear of side effects..1/0<br>स्वास्थ्य संबंधी समस्याएं/Health concerns ....1/0<br>पहुंच की कमी/ बहुत दूर/Lack of access s / too far......................................1/0<br>बहुत महंगी/Costs too much ....1/0<br>पसंदीदा तरीका उपलब्ध नहीं/Preferred method not available.....................1/0<br>कोई विधि उपलब्ध नहीं/No method available.......................................1/0<br>उपयोग करने में असुविधाजनक/Inconvenient to use ...1/0<br>शरीर की प्रक्रियाओं के साथ हस्तक्षेप/Interferes with body's processes ....1/0<br>अन्य/Other ....1/0<br>जानती नहीं/Don't know....................-88<br>कोई जवाब नहीं/No response............-99	319 = 0<br>AND<br>(212a OR 212b > 2 years) AND (211a = 2 OR 211b = 2)
323b	गर्भनिरोधक विधि का प्रयोग नहीं करना मुख्य रूप से आपका स्वयं का निर्णय था, या मुख्य रूप से आपके पति/साथी का या आप दोनों का संयुक्त निर्णय था?<br>**Would you say that not using contraception is mainly your decision, mainly your husband/partner's decision or did you both decide together?**	मुख्यतः उत्तरदाता का/Mainly respondent1<br>मुख्यतः पति या साथी का/Mainly husband/partner...............................2<br>सयुंक्त निर्णय था/Joint Decision.............3<br>अन्य/Other ....96<br>कोई जवाब नहीं/No response............-99	306a ≠ 1 AND 302a ≠ 1 AND LCL301 ≠ 1

324	पिछले 12 महीनों में, परिवार नियोजन के बारे में आप से कोई आंगनवाड़ी कार्यकर्ता, आशा (ASHA), या अन्य सामुदायिक स्वास्थ्य कार्यकर्ता द्वारा बात की गयी?<br>**In the last 12 months, were you visited by a anganwadi worker, ASHA, or other community health worker who talked to you about family planning?**	हाँ/Yes..1<br>नहीं/No..0<br>कोई जवाब नहीं/No response...........-99	009a = 1
325a	पिछले 12 महीनों के दौरान, क्या आप स्वयं हेतु या अपने बच्चों हेतु किसी स्वास्थ्य सेवा या कैम्प में गयीं?<br>**In the last 12 months, have you visited a health facility or camp for care for yourself or your children?**<br>*किन्हीं भी स्वास्थ्य सेवाओं के लिए*<br>*For any health services*	हाँ/Yes..1<br>नहीं/No..0<br>कोई जवाब नहीं/No response...........-99	009a = 1
325b	उस स्वास्थ्य सुविधा पर किसी भी स्टाफ सदस्य ने आपसे परिवार नियोजन के साधनों के बारे में बात की?<br>**Did any staff member at the health facility speak to you about family planning methods?**	हाँ/Yes..1<br>नहीं/No..0<br>कोई जवाब नहीं/No response...........-99	325a = 1

326	पिछले कुछ महीनों में आपने:<br>**In the last few months have you:**	हाँ/YES	नहीं/NO	कोई जवाब नहीं/NR	009a = 1
	रेडियो पर परिवार नियोजन के बारे में सुना है?<br>**a. Heard about family planning on the radio?**	1	0	-99	
	टेलीविजन पर परिवार नियोजन के बारे में कुछ भी देखा है?<br>**b. Seen anything about family planning on the television?**	1	0	-99	
	किसी अखबार या पत्रिका में परिवार नियोजन के बारे में पढ़ा है?<br>**c. Read about family planning in a newspaper or magazine?**	1	1	-99	
	मोबाईल फोन पर परिवार नियोजन के बारे में कोई वुड वोइस या लिखित संदेष प्राप्त किया है?<br>**d. Received a voice or text message about family planning on a mobile phone**	1	0	-99	

Section 4 - Sexual Activity

भाग 4 - यौन गतिविधियाँ

अन्य लोगों की उपस्थिति देखें। आगे बढ़ने से पहले एकान्त बनाने हेतु हर संभव प्रयास करें।

CHECK FOR THE PRESENCE OF OTHERS. BEFORE CONTINUING, MAKE EVERY EFFORT TO ENSURE PRIVACY.

401a	आपकी उम्र कितनी थी जब आपने पहली बार संभोग किया?<br>**How old were you when you first had sexual intercourse?**<br>उत्तरदाता पिछले जन्मदिन पर [age from 102] साल की थी।<br>***Current age: [AGE FROM 103]***<br>**[उसके x जीवित जन्म है]**<br>***Number of live births: [CALC_CEB]***<br>उम्र वर्ष में दर्ज करें।<br>अगर उत्तरदाता ने कभी संभोग नहीं किया, -77 दर्ज करें।<br>अगर उत्तरदाता को पता नहीं, -88 दर्ज करें।<br>अगर उत्तरदाता के पास कोई जवाब नहीं है, -99 दर्ज करें।<br>*Enter the age in years.*<br>*Enter -77 if she never had sex.*<br>*Enter -88 if respondent does not know.*<br>*Enter -99 for no response.*	उम्र/Age ☐	009a = 1
401b	आपने दर्ज किया कि उत्तरदाता X साल की थी जब उसने पहली बार संभोग किया था। क्या उसने यही कहा है?<br>**You have entered that the respondent was X years old when she first had sexual intercourse. Is this what she said?**<br>*अगर यह सही नहीं है, तो वापस 401a को सही कीजियें।*<br>*Go back and correct 401a if it is not correct.*	हाँ/Yes..1<br>नहीं/No 0	401a ≥ 0 AND 401a < 10 years and ≠ -77, -88 or -99
402	पिछली बार कब आपने संभोग किया?<br>**When was the last time you had sexual intercourse?**<br>*अगर 12 महीने से कम हैं, जवाब महीने, सप्ताह, या दिन में दर्ज किया जाना चाहिए।*<br>*आज के लिए 0 दिन दर्ज करें।*<br>*आप अगली स्क्रीन पर X के लिए एक नंबर दर्ज करेंगें।*<br>*If less than 12 months ago, answer must be recorded in months, weeks, or days.*<br>*Enter 0 days for today.*<br>*You will enter a number for X on the next screen.*	______ दिनों पहले/days ago<br>______ हफ़्तों पहले/weeks ago<br>______ महिनो पहले/months ago<br>______ सालों पहले/years ago	401a ≠ -77

Section 6 - Menstrual Hygiene<br>भाग- 6 माहवारी/मासिक धर्म सम्बंधित स्वच्छता<br><br>अब मैं आपसे मासिक धर्म सम्बंधित स्वच्छता के बारे में कुछ प्रश्न पूछूंगी। जिसमे आपके द्वारा इन दिनों में इस्तेमाल होने वाली सामग्री, एकांत, स्वच्छ, सुरक्षित स्थान की उपलब्धता धोने के लिए उचित प्रबंध एवं उपयोग में लायी सामग्री के निस्तारण के लिए स्थान से सम्बंधित प्रश्न होंगे। *Now I'm going to ask you about menstrual hygiene management. This includes the use of absorbent materials; access to a private, clean, safe space; washing as required; and a place to dispose used materials.*			
601	पिछली बार जब आपको माहवारी आई थी तो वे स्थान जहाँ पर आपने मासिक धर्म सम्बन्धी सामग्री को धोया, सुखाया, इस्तेमाल किया, व निस्तारण किया कौनसे थे?<br>**The last time you had your period, where are all the places where you changed, washed, dried, or disposed of used sanitary materials?**<br><br>गहराई से पूछें: कहीं और?<br>PROBE: Anywhere else?<br>विकल्पों को जोर से न पढ़ें। लागू होने वाले सभी विकल्प चुनें।<br>*Do not read options aloud. Select all that apply.*	[Main sanitation facility from HQ] .. 1/0<br>परिवार प्रश्नावली से चुनी गयी मुख्या शौचालयी सुविधा<br>Other household sanitation facility 1/0<br>परिवार में किसी अन्य प्रकार की शौचालय सुविधा<br>Sanitation facilities at school......... 1/0<br>विद्यालय में शौचालय सुविधाएं<br>Sanitation facilities at work .... 1/0<br>कार्य स्थल पर शौचालय सुविधाएं<br>Other public sanitation facility .... 1/0<br>अन्य सार्वजनिक शौचालय सुविधा<br>Sleeping area/bedroom .... 1/0<br>सोने के स्थान या शयनकक्ष<br>Backyard....................................... 1/0<br>घर के पिछवाड़े में<br>No facility/Bush/Field .... 1/0<br>कोई सुविधा नहीं/झाडी या खेत<br>Other... 1/0<br>अन्य<br>No response .... -99<br>कोई जवाब नहीं	(209 ≤ 90 days, 13 weeks or ≤ 3 months) AND 210 ≠ 1

602a	आप मासिक धर्म के दौरान उपयोग किये गए पैड्स, कपड़े या कोई अन्य मासिक धर्म सम्बन्धी सामग्री कहाँ बदलती हैं?<br>[ओ डी के प्रश्न *601* में चुनें गए विकल्पों को ही दिखायेगा]<br>**Where do you most often change your used pads, cloths, or other sanitary materials?**<br>*[ODK will only display the options selected in 601]*	[Main sanitation facility from HQ] .. 1/0<br>परिवार प्रश्नावली से चुनी गयी मुख्या शौचालयी सुविधा<br>Other household sanitation facility 1/0<br>परिवार में किसी अन्य प्रकार की शौचालय सुविधा<br>Sanitation facilities at school......... 1/0<br>विद्यालय में शौचालय सुविधाएं<br>Sanitation facilities at work .... 1/0<br>कार्य स्थल पर शौचालय सुविधाएं<br>Other public sanitation facility .... 1/0<br>अन्य सार्वजनिक शौचालय सुविधा<br>Sleeping area/bedroom .... 1/0<br>सोने के स्थान या शयनकक्ष<br>Backyard....................................... 1/0<br>घर के पिछवाड़े में<br>No facility/Bush/Field .... 1/0<br>कोई सुविधा नहीं/झाडी या खेत<br>Other... 1/0<br>अन्य<br>No response ....-99<br>कोई जवाब नहीं	More than 1 option selected in 601
602b	अपने मासिक-धर्म का प्रबंधन करते समय, क्या वह स्थानः<br>**While managing your menstrual hygiene, was this place:**<br>मुख्य स्थान: [601 या 602a में से चुने गए]<br>उत्तर के विकल्पों को पढ़कर सुनाए एवं सभी लागू उत्तरों को चुनें।<br>**Main place: [Selection from 601 or 602a]**<br>*Read each option aloud and select if yes.*	साफ़ था/Clean? .... 1/0<br>एकांत था/Private? .... 1/0<br>सुरक्षित था/Safe? .... 1/0<br>लॉक किया जा सकता था/Able to be locked? .... 1/0<br>साफ़ पानी की सुविधा युक्त था/Supplied with clean water? .... 1/0<br>साबुन की सुविधा युक्त था/Supplied with soap? .... 1/0<br>उपरोक्त में से कोई नहीं/None of the above ....-77<br>कोई जवाब नहीं/No response............-99	601≠-99 OR no facility and 602≠-99 OR no facility

603	**पिछली माहवारी के दौरान हुए रक्तस्त्राव को सोखने के लिए आपने किस सामग्री का उपयोग किया था?**<br>**During your last menstrual period, what did you use to collect or absorb your menstrual blood?**<br><br>गहराई के जांचें: कुछ और?<br>PROBE: Anything else?<br><br>विकल्पों को ज़ोर से न पढ़ें. लागू होने वाले सभी विकल्पों को चुनें।<br>*Do not read options aloud. Select all that apply.*	Reusable materials<br>दोबारा काम में आने वाली सामग्री<br>Cloth/Pieces of fabric............ 1/0<br>कपड़ा/कपड़े का टुकड़ा<br>Cotton wool........................... 1/0<br>रुई<br>Sanitary pads........................ 1/0<br>सेनिटरी पैड्स<br>Foam (from a mattress or other material)................................ 1/0<br>स्पंज (गद्दे या किसी अन्य वस्तु से निकला गया)<br>Other.................................... 1/0<br>अन्य<br>Disposable materials<br>उपयोग करके फेकनें वाली सामग्री<br>Tampons............................... 1/0<br>टेमपून<br>Toilet paper........................... 1/0<br>टॉयलेट पेपर<br>Paper (newspaper, pages from books).................................. 1/0<br>कागज़ (अख़बार या किताबों से)<br>Natural materials (mud, dung, leaves).................................. 1/0<br>प्राकृतिक सामग्री (मिट्टी, गोबर, पत्तियां)<br>No materials used...................... -77<br>कोई सामग्री इस्तमाल नहीं करते<br>No response ....-99<br>कोई जवाब नहीं	009a = 1
604a	क्या आपने अपनी पिछली माहवारी के दौरान पैड्स, कपड़े या अन्य मासिक धर्म सम्बंधित सामग्री को धोकर वापस प्रयोग में लिया था?<br>**Did you wash and reuse pads, cloths, or other sanitary materials during your last menstrual period?**	Yes...1<br>हाँ<br>No ....0<br>नहीं<br>No response ....-99<br>कोई जवाब नहीं	603=Reusable materials
604b	आपके पिछले मासिक-धर्म के दौरान जो पैड्स, कपड़े या अन्य मासिक-धर्म सामाग्री आपने धोकर पुनः प्रयोग की थी क्या वह हर बार पुनः प्रयोग करने से पहले पूरी तरहं से सूखी थी?<br>**During your last menstrual period, were the sanitary materials that you washed and reused completely dried before each reuse?**	हाँ/Yes...1<br>नहीं/No ....0<br>कोई जवाब नहीं/No response............-99	604a=1

605	आपके अनुसार आपने अपने गत मासिक-धर्म के दौरान [FQ603 के उत्तर] प्रयोग किया/किए थे। आपने इन सामग्री को प्रयोग करने के बाद इनका निपटारा कहां किया?<br><br>**You mentioned that you used [RESPONSES FROM FQ603] during your last menstrual period. Where did you dispose of these materials after use?**<br><br>गहराई से पूछेंः कहीं और?<br><br>उत्तर के विकल्पों को पढ़कर नहीं सुनाएं। सभी लागू उत्तरों को चुनें।<br><br>PROBE: Anywhere else?<br><br>*Do not read options aloud. Select all that apply.*	फ्लश शौचालय में/Flush toilet............1/0<br>लेट्रिन में/Latrine..............................1/0<br>कचरा पात्र/बैग मीम/Waste bin/Trash bag ....1/0<br>जलाया/Burning...............................1/0<br>झाड़ियों/खेतों में/Bush/Field ....1/0<br>अन्य/Other......................................1/0<br>कोई जवाब नहीं/No response............-99	603=Disposable material s OR 604a=0
606	क्या कोई ऐसी बात/वस्तु है जो आपको मासिक-धर्म के प्रबंधन में मददगार हो सकती है लेकिन आप सामान्यतः उसका लाभ नहीं ले पाती?<br>**Is there anything else that would help you manage your menstrual period that you do not usually have?**<br><br>गहराई से पूछें: कुछ और?<br><br>सुझाव: इसमें संसाधन, सामाग्री, आपके वातावरण में बदलाव, आदि को शामिल कर सकते हैं।<br><br>PROBE: Anything else?<br><br>*Hints: Could include resources, materials, changes to your environment, etc. Do not read options aloud.*	मेरे पास सब आवश्यक चीज़ें/सुविधाएं हैं/ I have all I need ....1/0<br>साफ़ पानी/ Clean water...................1/0<br>साबुन/Soap.....................................1/0<br>स्वच्छ अवशोषण सामग्री/Clean absorbent materials ....1/0<br>एक निजी स्थान/A private place ....1/0<br>एक सुरक्षित स्थान/A place where I feel safe ....1/0<br>और जानकारी/More knowledge ....1/0<br>स्वच्छ अव्शेशक खरीदने का एक स्थान/ A place to buy clean absorbent materials ....1/0<br>उपयोग किये गए सामान को सुखाने का एक स्थान /A place to dry used materials1/0<br>उपयोग किये गए सामान को निपटाने का एक स्थान /A place to dispose used materials ....1/0<br>पैसा/Money.....................................1/0<br>दर्द का इलाज/Pain medication..........1/0<br>अन्य/Other......................................1/0<br>कोई जवाब नहीं/No response............-99	009a=1

END OF SURVEY
साक्षात्कार की समाप्ति
Thank the respondent for her time
उत्तरदाता को उनके द्वारा दिये गये समय के लिए धन्यवाद दें।
उत्तरदाता का भाग समाप्त हो गया है, लेकिन अभी 3 प्रश्न हैं जिन्हें आपको घर के बाहर जाकर पूरा करना है।
The respondent is finished, but there are still 3 more questions for you to complete outside the home.

095	स्थान<br>**Location**<br>*घर के प्रवेश द्वार के पास एक जी.पी.एस बिन्दु लें या स्थान रिकार्ड करें जब एक्यूरेसी 6 मीटर से कम हो।*<br>जी.पी.एस. के कोरडीनेट्स केवल बाहर जाने पर ही लिए जा सकते हैं।<br>*Take a GPS point near the entrance to the household. Record location when the accuracy is smaller than 6m.*<br>*GPS coordinates can only be collected when outside.*	स्थान/LOCATION	Always
096	कितनी बार आपने इस महिला उत्तरदाता के साक्षात्कार के लिए इस घर का दौरा किया?<br>**How many times have you visited this household to interview this female respondent?**	पहली बार/1st time..............................1<br>दूसरी बार/2nd time ....2<br>तीसरी बार/3rd time 3	Always
097	साक्षात्कार किस भाषा में किया गया है?<br>**In what language was this interview conducted?**	English ....1<br>Hindi..3<br>Other...96	009a=1
098	प्रश्नावली परिणाम<br>**Questionnaire result**<br>महिला प्रश्नावली का परिणाम दर्ज करें।<br>*Record the result of the Female Questionnaire*	पूरा हुआ/Completed ....1<br>घर पर नहीं/Not at home ....2<br>स्थगित/Postponed ....3<br>इनकार किया/Refused ....4<br>आंशिक रूप से पूरा/Partly completed.....5<br>अक्षम/Incapacitated 6	Always
